# Carbon and Nitrogen Allocation between the Sink and Source Leaf Tissue in Response to the Excess Excitation Energy Conditions

**DOI:** 10.3390/ijms24032269

**Published:** 2023-01-23

**Authors:** Dejana Milić, Bojana Živanović, Jelena Samardžić, Nenad Nikolić, Caroline Cukier, Anis M. Limami, Marija Vidović

**Affiliations:** 1 University of Belgrade, Institute of Molecular Genetics and Genetic Engineering, Laboratory for Plant Molecular Biology, Vojvode Stepe 444a, 11042 Belgrade, Serbia; 2 Institute for Multidisciplinary Research, University of Belgrade, Kneza Višeslava 1, 11000 Belgrade, Serbia; 3 Univ Angers, INRAE (Institut National de Recherche pour l’Agriculture, l’Alimentation et l’Environnement), 49000 Angers, France

**Keywords:** ^15^N nitrate labelling, cell wall, high light intensity, free amino acids, phenolic compounds, *Pelargonium zonale*, variegated plants

## Abstract

Plants are inevitably exposed to extreme climatic conditions that lead to a disturbed balance between the amount of absorbed energy and their ability to process it. Variegated leaves with photosynthetically active green leaf tissue (GL) and photosynthetically inactive white leaf tissue (WL) are an excellent model system to study source–sink interactions within the same leaf under the same microenvironmental conditions. We demonstrated that under excess excitation energy (EEE) conditions (high irradiance and lower temperature), regulated metabolic reprogramming in both leaf tissues allowed an increased consumption of reducing equivalents, as evidenced by preserved maximum efficiency of photosystem II (*Ф_PSII_*) at the end of the experiment. GL of the EEE-treated plants employed two strategies: (i) the accumulation of flavonoid glycosides, especially cyanidin glycosides, as an alternative electron sink, and (ii) cell wall stiffening by cellulose, pectin, and lignin accumulation. On the other hand, WL increased the amount of free amino acids, mainly arginine, asparagine, branched-chain and aromatic amino acids, as well as kaempferol and quercetin glycosides. Thus, WL acts as an important energy escape valve that is required in order to maintain the successful performance of the GL sectors under EEE conditions. Finally, this role could be an adaptive value of variegation, as no consistent conclusions about its ecological benefits have been proposed so far.

## 1. Introduction

Climate change brings fluctuating and extreme environmental conditions, such as high solar radiation, low and high temperatures, drought and increased CO_2_ levels [1]. The effects of these factors on plants are often interrelated and usually result in a disturbed balance between the amount of received energy and the ability to process it [2,3]. A high intensity of photosynthetically active radiation (PAR) increases the excitation pressure in chloroplasts and accelerates the generation of reactive oxygen species (ROS), which can lead to cellular redox imbalance and reduced plant growth and productivity [1,4]. On the other hand, redox and ROS signals originating from photosynthesising chloroplasts (such as the ratio of reduced plastoquinone, hydrogen peroxide and oxidised chloroplastic metabolites) can control the nuclear gene expression related to primary and secondary metabolism, independently or in cooperation with light signals in the cytosol via an unknown mechanism [5,6].

To maintain energy balance, the additional electron sinks are required to efficiently dissipate excess excitation energy (EEE) and keep the pools of ATP/ADP, NAD (P)H/NAD (P) and other redox carriers in equilibrium [7]. This can be achieved through non-photochemical and photochemical pathways such as photorespiration, the water–water cycle, polyphenol biosynthesis, and nitrite reduction [2,8,9]. However, there are no consistent conclusions on the influence of nitrate reduction and nitrogen allocation on photosynthetic photoprotection. The increased PAR intensity and low temperatures direct carbon allocation towards a higher C/N ratio through the stimulated biosynthesis of phenols as N-free compounds, which can also be considered as an energy escape valve [10]. The involvement of phenolics in plant–environment interactions is well known, but their tissue-specific accumulation under certain abiotic stresses opens a debate on their physiological functions (UV/light attenuation, an antioxidant, or a sink for reduced carbon) [11,12,13].

The phenylpropanoid and flavonoid glycosides and derivatives predominantly accumulate in the vacuoles and cell walls of the epidermal and guard cells. They can attenuate UV-B and UV-A radiation, while anthocyanins strongly absorb about 520 nm and protect the photosynthetic apparatus from excess PAR and potential photo-oxidative stress [12]. On the other hand, flavonoid accumulation has also been found in the vacuoles and chloroplasts of mesophyll cells [14]. Flavonoids with anthocyanidins and hydroxycinnamic acids (HCAs) are endogenous substrates for vacuolar and apoplastic class III peroxidases (PODs) and are involved in H_2_O_2_ scavenging together with ascorbate [15,16]. *Ortho*-dihydroxy-B-ring-substituted flavonoids have been detected in the chloroplast envelope and nucleus of mesophyll cells of various plant species, and are thought to be able to protect thylakoids and photosynthetic machinery from photo-oxidative damage by directly scavenging ROS and preventing lipid peroxidation [17]. Monolignols originate from the phenylpropanoid pathway and their polymerisation leads to lignin formation. Together with pectin, hemicellulose, cellulose, and structural proteins, lignin is an important component of the cell wall, responsible for its mechanical strength, rigidity and hydrophobicity [18].

Leaf variegation (e.g., in *Pelargonium zonale*) has proven to be a suitable model system to study source–sink interactions in terms of carbon and nitrogen allocation within the same leaf under the same microenvironmental conditions [19,20]. These leaves contain photosynthetically inactive white leaf tissue (WL) at the leaf margin and photosynthetically active green leaf tissue (GL) in the leaf centre. These two metabolically contrasting tissues have shown different subcellular distributions of low-molecular-weight antioxidants (ascorbate and glutathione) and different distributions of phenolic compounds, antioxidants (enzymatic and non-enzymatic), soluble sugars, and H_2_O_2_ under optimal growth conditions [21,22]. Moreover, GL and WL have shown a different response of antioxidant phenolic metabolism to UV-B radiation and high PAR intensities [20]. Furthermore, these two tissues have shown different antioxidant responses under conditions that accelerate the Mehler reaction (high PAR, paraquat) in chloroplasts [22].

In our previous work [19], the differential transcriptomic analysis of the gene expression of *P. zonale* GL and WL under optimal growth conditions was performed for the first time. It was found that the WL tissue had a higher content of nitrogenous compounds, especially free amino acids (AAs), compared to GL. Moreover, WL possessed the enzymatic arsenal to synthesise all of the proteogenic AAs. Tcherkez [23] and co-workers showed that variegated *P. zonale* morphs tolerated nitrogen deprivation better than plain morphs due to the remobilisation of nitrogen from the white to the green leaf sectors. Indeed, more than half of the identified transcripts encoding various amino acid transporters were more highly expressed in WL than in GL [19].

The aim of this study is to clarify the influence of additional sink tissue within the same leaf on the photosynthetic rate and photoinhibition at high PAR intensity in variegated *P. zonale*. The influence of alternative sinks on the enhancement of photoinhibition should be re-examined with respect to newly investigated alternative pathways of electron partitioning in photosynthesis [6]. The advantage of using variegated leaves is the possibility of investigating nitrogen and carbon allocation between autotrophic and heterotrophic tissues within the same plant organ, in contrast to the usual shoot–root studies. We hypothesised that the metabolism of the WL sectors serves as a potential energy escape valve required to maintain the successful performance of the GL sectors under high-irradiance conditions. Furthermore, we aimed to investigate the physiological role of accumulated AAs and phenolic compounds in WL under high light as alternative electron sinks for linear photosynthetic electron transfer. To achieve these objectives, we compared WL and GL of *P. zonale* plants under low light (LL) and high light (HL) conditions at two temperatures (18 °C and 25 °C) and analysed photosynthetic and morphological traits, carbon (polyphenols) and nitrogen (AAs) allocation, and the expression of the related genes. Using the stable-isotope-labelled substrates such as [^15^N] KNO_3_ provided further insight into AA regulation in these two tissues under elevated EEE conditions. 

## 2. Results

### 2.1. Effect of EEE on the Chlorophyll Fluorescence Parameters

**Figure 1:**
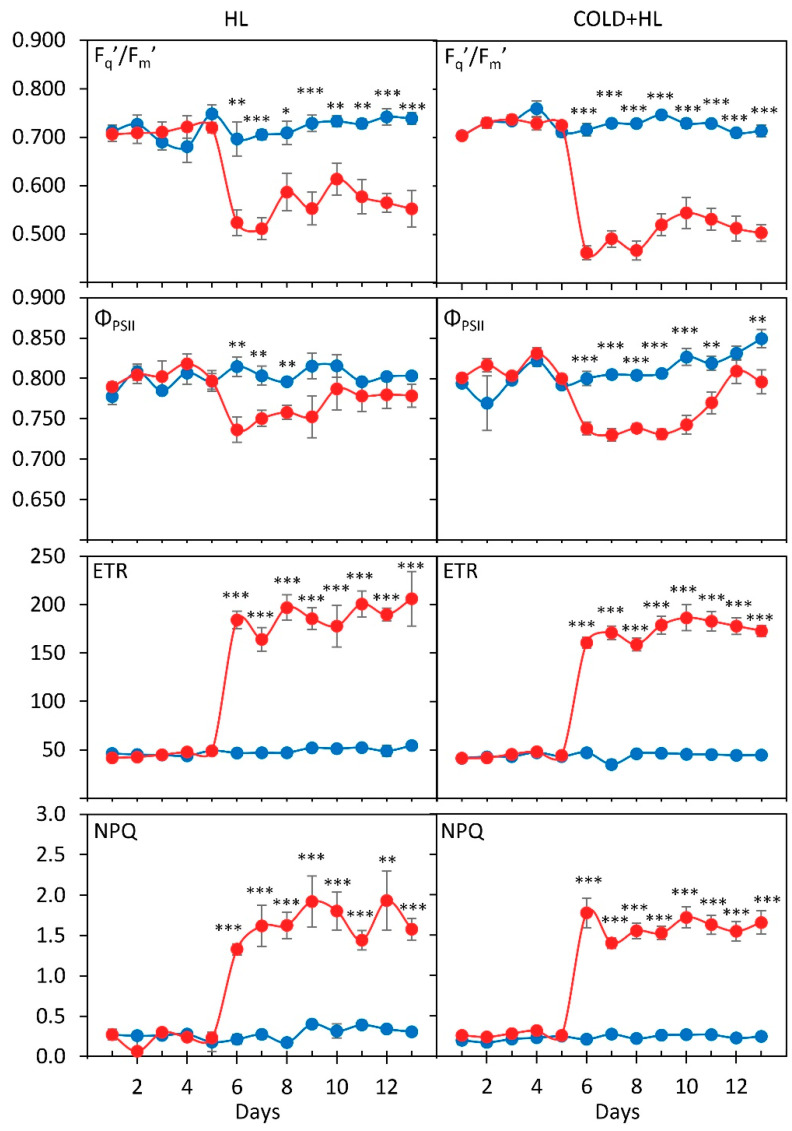
Operating efficiency PS II (F_q_′/F_m_′), maximum efficiency PS II (*Φ*_PSII_, F_v_/F_m_), electron transfer rate (ETR), and non-photochemical quenching (NPQ) of GL of *P. zonale* plants during a five-day pretreatment (LL) and an eight-day exposure to low light (LL, 180 µmol m^–2^ s^–1^, blue line) and to high light (HL, 800 µmol m^–2^ s^–1^, red line) in the *HL* (25 °C; left) and *Cold + HL* (18 °C; right) experiments. Values represent means ± SE (*HL*: *n* = 10; *Cold + HL*: *n* = 22). Significant differences between plants treated with LL and HL, according to the *t*-test, are indicated in each experiment (* *p* < 0.05; ** *p* < 0.01; *** *p* < 0.001).

As expected, the operating efficiency of photosystem II (PS II) - F_q_′/F_m_′ decreased more in plants exposed to HL (800 µmol m^–2^ s^–1^) than in those exposed to LL (180 µmol m^–2^ s^–1^) during the eight days in both experiments (Figure 1, significant “PAR” effects, Supplementary Table S1). The extent of the F_q_′/F_m_′ decline under HL was greater in the *Cold + HL* experiment than in the *HL* experiment. In LL-treated plants, F_q_′/F_m_′ was more stable during the experiment, while it varied under HL during exposure at both temperatures. HL induced a statistically significant decrease in the maximum PS II efficiency, *Φ*_PSII_, already on the first day (Figure 1). However, on the fifth day of HL exposure in the *HL* experiment, and on the seventh day in the *Cold + HL* experiment, the *Φ*_PSII_ values approached those measured before treatment (the fifth day, statistically significant).

Non-photochemical quenching (NPQ) was 3.7–9.4 times higher under HL at 25 °C than in LL-exposed leaves, while this ratio was 5.9–8.5 at 18 °C (Figure 1, significant “PAR” effects, Supplementary Table S1).

The trends of all four measured parameters were similar in both experiments and no statistical differences were found in the parameter changes as a function of temperature.

### 2.2. Effect of EEE on Epidermal Flavonoid and Chlorophyll Contents in P. zonale Leaves

**Figure 2:**
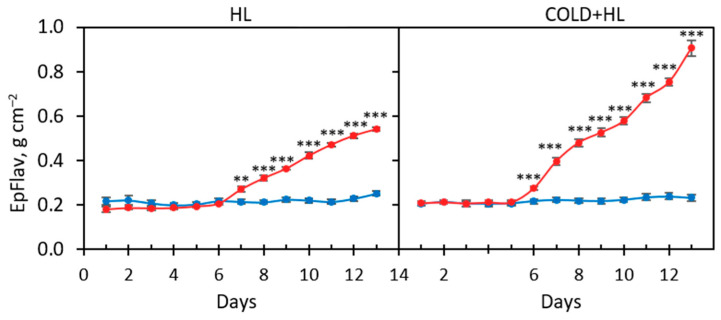
The change in the content of the epidermal flavonoids (EpFlav) in the GL adaxial epidermis of *P. zonale* plants during a five-day pretreatment (LL) and an eight-day exposure to low light (LL, 180 µmol m^–2^ s^–1^, blue line) and to high light (HL, 800 µmol m^–2^ s^–1^, red line) in the *HL* (25 °C; left) and *Cold + HL* (18 °C; right) experiments. Values represent means ± SE (*HL*: *n* = 10; *Cold + HL*: *n* = 22). Significant differences between plants treated with LL and HL, according to the *t*-test, are indicated in each experiment (** *p* < 0.005, *** *p* < 0.001).

The rapid accumulation of epidermal flavonoids (EpFlav) in *P. zonale* leaves was observed as early as the first day after HL exposure at 18 °C and on the second day at 25 °C and continued to increase with increasing duration of HL exposure (Figure 2, significant “day” and “PAR” effects, Supplementary Table S2). The total accumulation of EpFlav was more pronounced in the *Cold + HL* experiment than in the *HL* experiment. At the end of the experiment, the EpFlav content in the plants used in the *Cold + HL* experiment was more than 60% higher than in the *HL* experiment.

**Figure 3:**
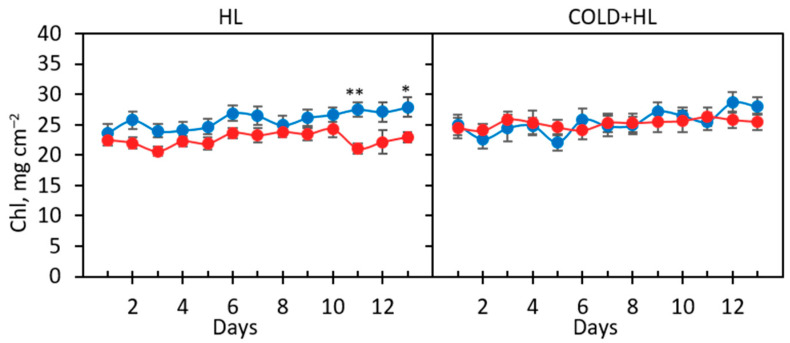
Chlorophyll content in the GL adaxial epidermis of *P. zonale* plants during a five-day pretreatment (LL) and an eight-day exposure to low light (LL, 180 µmol m^–2^ s^–1^, blue line) and to high light (HL, 800 µmol m^–2^ s^–1^, red line) in the two experiments *HL* (25 °C; left) and *Cold + HL* (18 °C; right). Values represent means ± SE (*HL*: *n* = 10; *Cold + HL*: *n* = 22). Significant differences between plants treated with LL and HL, according to the *t*-test in each experiment are indicated (* *p* < 0.05, ** *p* < 0.005).

In parallel, the amount of chlorophyll (Chl) did not change significantly between the LL- and HL-treated plants during the eight-day *Cold + HL* experiment, although a decreasing trend was observed (Figure 3; a significant “PAR” effect was observed only in the *HL* experiment, Supplementary Table S2). However, it decreased significantly in the last three days of the *HL* experiment (Figure 3). No significant change in Chl content as a function of temperature was observed.

### 2.3. Effect of EEE Conditions on Phenolic Content

**Figure 4:**
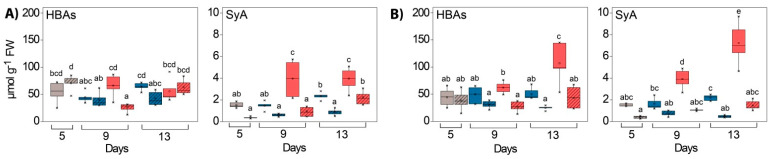
Changes in the composition of total hydroxybenzoic (HBA) and syringic acid (SyA) in GL (solid boxes) and WL (striped boxes) of *P. zonale* plants before (5 d, grey) and after four (9 d) and eight days (13 d) of high light exposure (HL, 800 µmol m^–2^ s^–1^, red bars) compared to plants exposed to low light (LL, 180 µmol m^–2^ s^–1^, blue bars) at (**A**) optimal temperature of 25 °C (*HL*) and (**B**) lower temperature of 18 °C (*Cold + HL*). Values are shown in µmol g^–1^ FW ± SE, *n* = 5–9. Different letters indicate statistically significant differences between different PAR intensities, time points, and leaf tissues (*p* < 0.05) according to Tukey’s post hoc test.

The composition of phenolic compounds was generally the same in GL and WL tissues but differed quantitatively (“tissue” effects, Supplementary data, Table S3). The profile of polyphenols found in our cultivar (including gallic, protocatechuic, *p*-coumaric and caffeic acid, as well as quercetin and kaempferol) was similar to that of *P. zonale* cv. “Ben Franklin” and other *P. zonale* species, as already shown in Milić et al. [19]. In both tissue types, hydroxybenzoic acids (HBAs) were the most abundant polyphenol class, with the highest content of syringic acid (SyA). At the beginning of both experiments, the concentrations of HBAs measured in GL and WL were similar (Figure 4).

**Figure 5:**
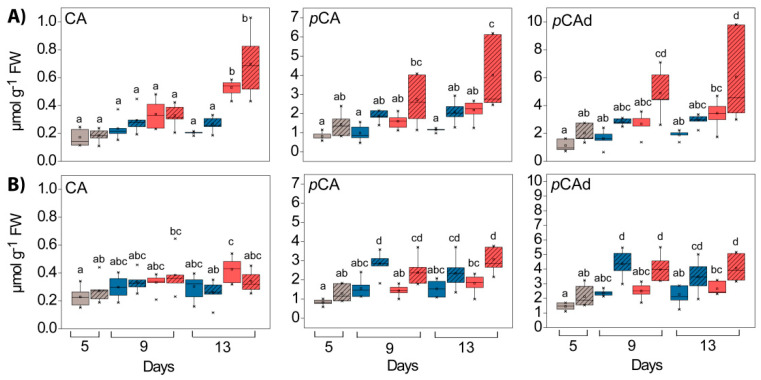
Changes in the composition of hydroxycinnamates in GL (solid boxes) and WL (striped boxes) of *P. zonale* plants before (5 d, grey) and after four (9 d) and eight days (13 d) of high light exposure (HL, 800 µmol m^–2^ s^–1^, red bars) compared to plants exposed to low light (LL, 180 µmol m^–2^ s^–1^, blue bars) at (**A**) optimal temperature of 25 °C (*HL*) and (**B**) lower temperature of 18 °C (*Cold + HL*). Values are shown in µmol g^–1^ FW ± SE, *n* = 5–9. Different letters indicate statistically significant differences between different PAR intensities, time points, and leaf tissues (*p* < 0.05) according to Tukey’s post hoc test. CA, caffeic acid; *p*-CA, *p*-coumaric acid; *p*-CAd, *p*-coumaric acid derivative.

Among the HCAs, derivatives (preferably glycosides and esters) of caffeic acid (CA) and *p*-coumaric acid (*p*-CA) were observed. CA was similarly distributed in GL and WL at the beginning of both experiments, while *p*-CA was slightly more abundant in WL than in GL (Figure 5). After the HL exposure, the levels of CA and *p*-CA increased in both tissues, particularly in WL, which was exposed to HL (Figure 5, “PAR” effect in Supplementary Table S3).

**Figure 6:**
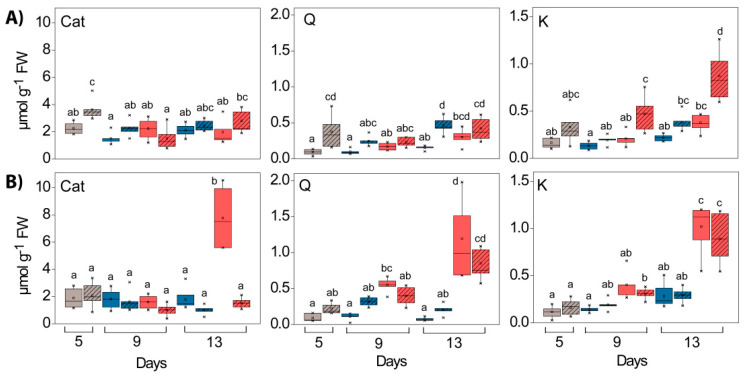
Changes in the composition of flavan-3-ols (catechin, Cat) and flavon-3-ols (quercetin, Q and kaempferol, K) in GL (solid boxes) and WL (striped boxes) of *P. zonale* plants before (5 d, grey) and after four (9 d) and eight days (13 d) of high light exposure (HL, 800 µmol m^–2^ s^–1^, red bars) compared to plants exposed to low light (LL, 180 µmol m^–2^ s^–1^, blue bars) at (**A**) optimal temperature of 25 °C (*HL*) and (**B**) lower temperature of 18 °C (*Cold + HL*). Values are shown in µmol g^–1^ FW ± SE, *n* = 5–9. Different letters indicate statistically significant differences between different PAR intensities, time points, and leaf tissues (*p* < 0.05) according to Tukey’s post hoc test.

Before HL exposure, the catechin (Cat) content was slightly higher in WL compared to GL (not statistically relevant), and in contrast to the *HL* experiment at 25°, it increased significantly (four times) in *Cold + HL* after eight days of exposure to higher EEE only in GL (Figure 6, “PAR” and “tissue” effects and their interactions in Supplementary Table S3).

In both tissue types, the flavone-3-ols quercetin (Q) and kaempferol (K) were present as mono- and diglycosides containing glucose, galactose, and rhamnose [19]. After hydrolysis, their concentrations in WL were slightly increased compared to GL in both experiments on the fifth day (Figure 6, “tissue” effects in Supplementary Table S3). However, when exposed to HL their content increased significantly, especially in combination with the lower temperature (Figure 6, “PAR” and “tissue” effects and their interactions in Supplementary Table S3). In contrast to the *Cold + HL* experiment, in the *HL* experiment after eight days of exposure to HL, the K content was significantly higher in WL than in GL (Figure 6).

### 2.4. Morphological Changes in P. zonale Leaves Induced by EEE Conditions

**Figure 7:**
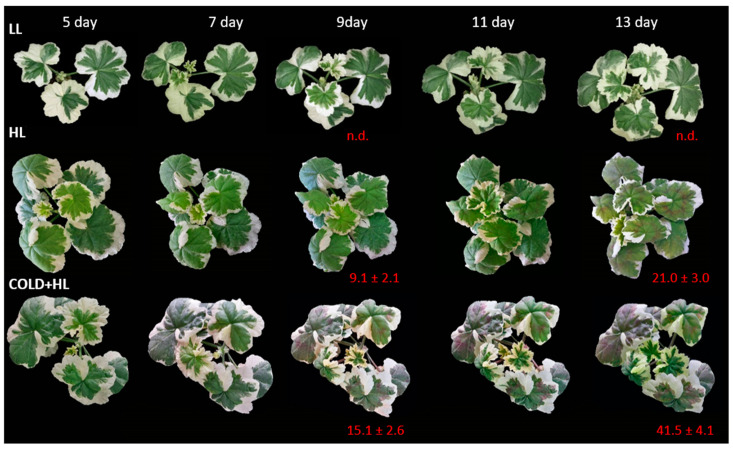
Representative *P. zonale* plants and cyanidin (Cy) concentrations (nmol g^–1^ FW ± SE, *n* = 5–9) after acid hydrolysis in GL during the *HL* and *Cold + HL* experiments.

EEE induced two morphological changes in *P. zonale* leaves: (i) the development of red colouration due to anthocyanin accumulation exclusively in GL and (ii) leaf wrapping and firming (Figure 7, Supplementary Figure S1). This was more pronounced in plain morphs, where anthocyanin accumulation was also observed in the lower epidermis. The accumulation of anthocyanins (mainly cyanidin glycosides) in the leaves was observed as early as the second day of HL exposure, regardless of temperature (Figure 7). However, the intensity of HL-induced cyanidin doubled at a lower temperature in the *Cold + HL* experiment (Supplementary Figure S1).

**Figure 8:**
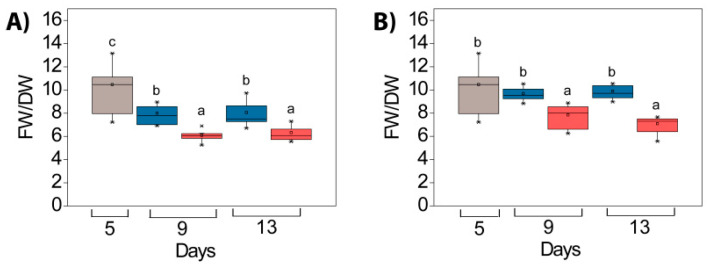
Fresh and dry weight ratio (FW/DW) in *P. zonale* leaves before (5 d, grey) and after four (9 d) and eight days (13 d) of high light exposure (HL, 800 µmol m^–2^ s^–1^, red bars) compared to plants exposed to low light (LL, 180 µmol m^–2^ s^–1^, blue bars) at (**A**) optimal temperature of 25 °C (*HL*) and (**B**) lower temperature of 18 °C (*Cold + HL*). Values are shown in µmol g^–1^ FW ± SE, *n* = 6–15. Different letters indicate statistically significant differences between different PAR intensities, time points, and leaf tissues (*p* < 0.05) according to Tukey’s post hoc test.

In both experiments (*HL* and *Cold + HL*), the ratio of fresh weight to dry weight (FW/DW) of *P. zonale* leaves (both tissue types) was lower in the HL-treated plants than in the LL-treated plants, already after four days (Figure 8, significant “PAR” effects, Supplementary Table S4). This ratio did not change significantly in the second half of the experiments (independent of temperature).

As plants have a higher transpiration rate under HL than under LL, the relative water content (RWC) was measured. Leaves exposed to LL had a slightly lower RWC (84.3 ± 4.3) than plants exposed to HL (93.4 ± 0.4), with no statistical differences in either experiment. Leaf density (ratio of DW to leaf area) did not differ significantly between these two light intensities.

### 2.5. Effects of EEE Conditions on the Cell Wall Components of P. zonale Leaves

The observed changes in leaf morphology and the decrease in the FW/DW ratio in leaves exposed to EEE encouraged us to evaluate possible changes in the cell wall constituents of these two leaf tissue types.

**Figure 9:**
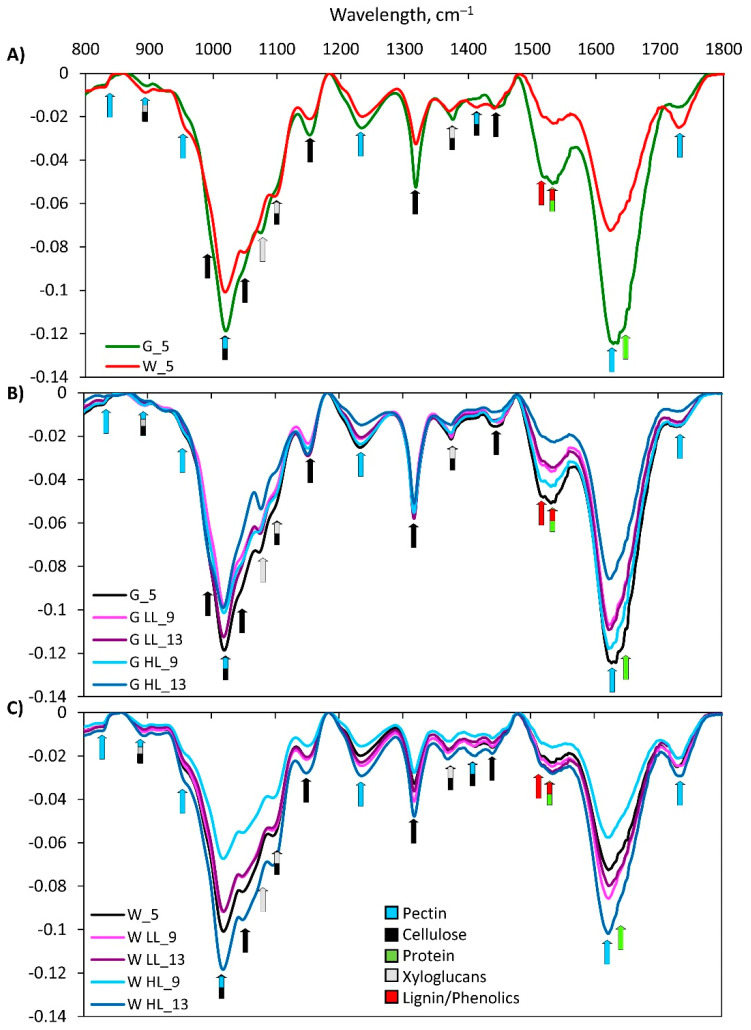
FTIR spectra of cell walls of (**A**) GL (green line) and WL (red line) *P. zonale* grown under LL; (**B**) GL and (**C**) WL at the beginning (5 d) and after four (9 d) and eight days (13 d) of exposure under HL and lower temperature (18 °C, *Cold + HL*). For each spectrum, the average spectra of at least three GL and WL samples (biological replicates) are shown. The peaks of cellulose, pectin, xyloglucan, proteins, and lignin (phenolic ring and phenolic esters) are highlighted. Cellulose: 898, 1005, 1020, 1052, 1097, 1147, 1320, 1376, 1416, 1443 cm^–1^; pectin: 822, 899, 955, 1017, 1236, 1420, 1630, 1733 cm^–1^; xyloglucan: 898, 1072, 1097, 1147, 1368 cm^–1^; lignin: 1517, 1534 cm^–1^.

Fourier transform infrared spectroscopy (FTIR) was used to profile the plant cell walls. The FTIR spectra of the cell walls of GL and WL from the *Cold + HL* experiment are shown in Figure 9. The bands associated with cellulose, such as a symmetric CH_2_ vibration at 1440 cm^−1^, a symmetric bending vibration at 1416 cm^−1^, a CH_2_ bending vibration at 1368 cm^−1^, a CH_2_ wagging vibration at 1320 cm^−1^, an asymmetric O–C–O stretching vibration of the glycosidic bond at 1147 cm^−1^, 1105 C–O and C–C stretching vibrations, a C–OH stretching secondary alcohol vibration at 1052 cm^−1^, a C-OH stretching primary alcohol vibration at 1029 cm^−1^, and a C–C stretching vibration (C_6_–H_2_–O_6_) at 990 cm^−1^ were found in both cell wall samples (references can be found in [24]). The bands at 1733 cm^−1^ (C=O stretching vibration of alkyl ester), 1630 cm^−1^ (COO– antisymmetric stretching vibration of polygalacturonic acid), 1420 cm^−1^ (COO– stretching vibration), 1236 cm^−1^ (C–O stretching vibration), 1017 cm^−1^ (C–O, C–C, C_2_–C_3_, C_2_–O_2_, C_1_–O_1_ stretching vibrations), 955 cm^−1^ (CO bending vibration), and 822 cm^−1^ (C-ring vibration) are typical for pectin. The peaks at 1517 cm^−1^ and 1534 cm^−1^ are characteristic for lignin. The peak at 1650 cm^−1^ is related to the C–N amide 1 stretching vibration in proteins and the peak at 1150 cm^−1^ is an indication of C–N deformation in proteins (Figure 9). The bands at 898 cm^−1^, 1072 cm^−1^ (C–O and C–C stretching vibrations), 1020 cm^−1^ (C–OH stretching vibration of primary alcohol groups), and 1368 cm^−1^ are typical for xyloglucans [24].

**Figure 10:**
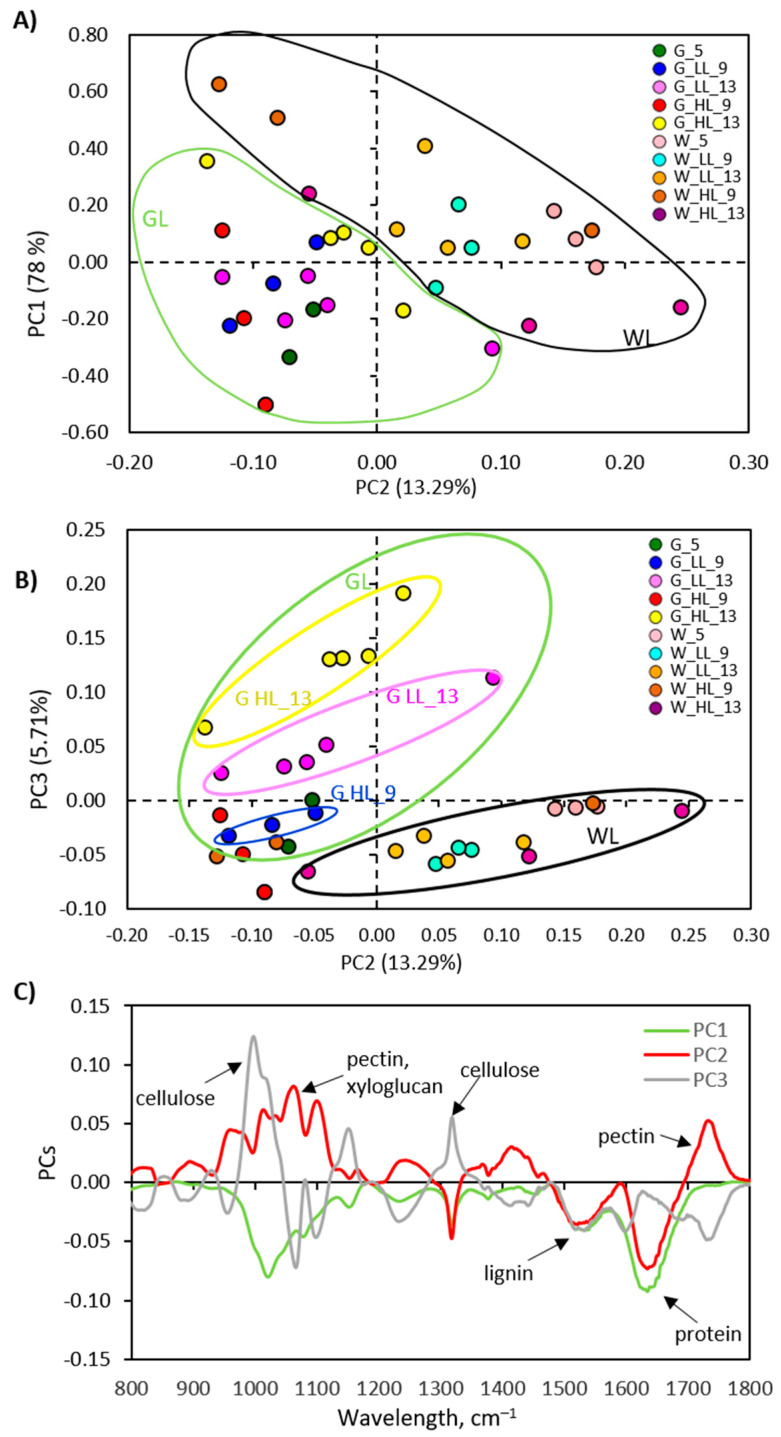
Graphical representation of the (**A**,**B**) scores and (**C**) loadings PC1, PC2, and PC3 determined using the spectral range 800–1800 cm^−1^ of GL and WL samples.

The effect of increased EEE on the content of specific components of the cell wall of *P. zonale* GL and WL in the *Cold + HL* experiment was analysed by using FTIR spectroscopy and principal component analysis (PCA). PCA was applied to a spectral range of 800–1800 cm^−1^ with the first principal component (PC1) explaining 78% of the total variance, the second (PC2) 13.3%, and the third 5.7% (Figure 10). The spectra shown in Figure 9 and PC1 (Figure 10A,C) indicate a generally higher content of cellulose, pectins, xyloglucans, proteins, and lignin in the GL cell wall than in the WL cell wall. Of all three PCs, PC2 best shows the difference between GL and WL, regardless of treatment (Figure 10A). This difference implies a lower content of pectin and hemicellulose (xyloglucans) and a higher amount of lignin, proteins, and cellulose in GL compared to WL (Figure 10C). PC3 illustrates the difference between GL after nine days of exposure to LL and 13 days of exposure to HL and the other treatment groups, including the WL tissue type (Figure 10B). PC3 loading revealed an increase in cellulose, as the dominant cell wall component compared to lignin, pectins, xyloglucans, and proteins in GL exposed to HL compared to LL on the 13th day (Figure 10C). According to the results obtained, the response of GL cell wall constituents was more intense than that of WL.

**Figure 11:**
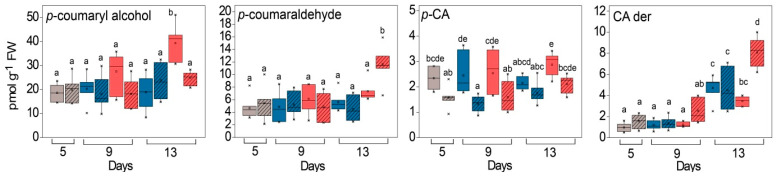
Content of cell-wall-bound hydroxycinnamates in GL (solid boxes) and WL (striped boxes) of *P. zonale* plants prior to (5 d, grey) and after four (9 d) and eight days (13 d) of exposure to high light (HL, 800 µmol m^–2^ s^–1^, red) compared to those exposed to low light (LL, 180 µmol m^–2^ s^–1^, blue) at lower temperature, 18 °C (*Cold + HL*). Values are shown in pmol g^–1^ FW ± SE, *n* = 7–9. Different letters denote statistically significant differences between different PAR intensities, time points, and leaf tissues (*p* < 0.05) according to Tukey’s post hoc test.; CA der, caffeic acid derivative with Rt = 22.5 min (Supplementary Figure S2).

To substantiate the previous results, the phenolic compounds bound to the cell wall were extracted and characterised. After alkaline hydrolysis, the most abundant compound was the monolignol, *p*-coumaryl alcohol (Figure 11). In addition, *p*-coumaric acid and *p*-coumaraldehyde, as well as unidentified *p*-CA and CA derivatives were detected. Before exposure to HL, the amount of *p*-coumaryl alcohol did not differ significantly between WL and GL (Figure 11). However, after exposure to HL in the *Cold + HL* experiment, the levels of *p*-coumaryl alcohol and *p*-coumaraldehyde tended to increase, more rapidly in GL (Figure 11, significant “PAR” effects, Supplementary Table S5). The amount of *p*-coumaryl alcohol doubled in GL after eight days of HL exposure.

### 2.6. Effect of EEE Conditions on the Free Amino Acid Content

In addition to the phenolic compounds described, the total and the individual AAs in both tissue types also responded to the increased EEE conditions. The total AA content was 1.2–3.5 times higher in WL compared to GL depending on the experimental conditions (Table 1, significant “tissue” effects, Supplementary Table S6). At the beginning of the experiment, the most abundant AAs in GL were Glu, Thr and Ala, while WL was richest in γ-aminobutyric acid (GABA), Asp and Thr (Supplementary Figure S2). The stimulatory effect of HL on the content of the total AA pool was observed in both tissues and at both temperatures, while the influence of GL was more pronounced at 25 °C (Table 1).

**Figure 12:**
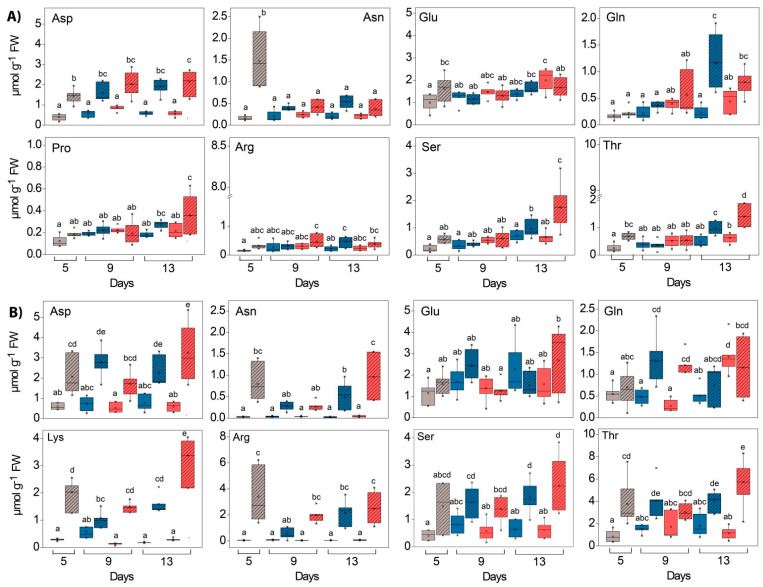
Content of polar free amino acids (Asp, Asn, Glu, Gln, Pro/Lys, Arg, Ser, Thr) in GL (solid boxes) and WL (striped boxes) of *P. zonale* plants prior to (5 d, grey) and after four (9 d) and eight days (13 d) of exposure to high light (HL, 800 µmol m^–2^ s^–1^, red) compared to those exposed to low light (LL, 180 µmol m^–2^ s^–1^, blue) under (**A**) optimal (25 °C, *HL*) and (**B**) lower (18 °C, *Cold + HL*) temperatures. Values are shown in µmol g^–1^ FW ± SE, *n* = 6–8. Different letters indicate statistically significant differences between different PAR intensities, time points, and leaf tissues (*p* < 0.05) according to Tukey’s post hoc test.

**Figure 13:**
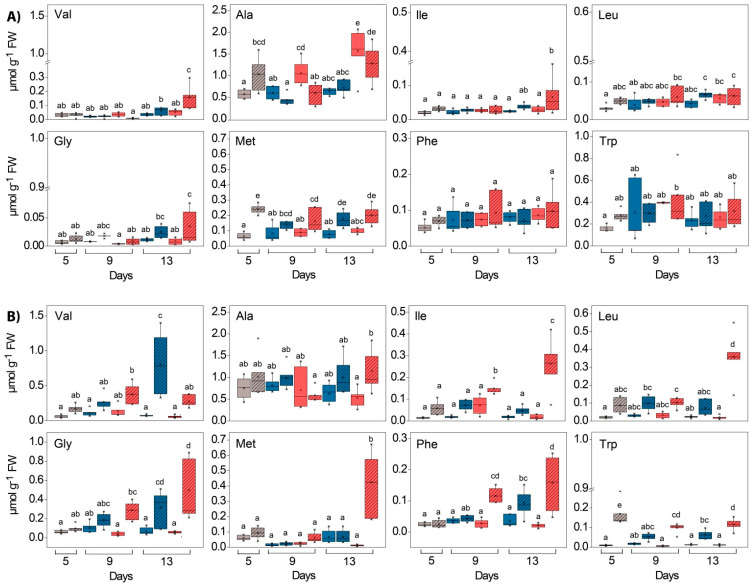
Content of nonpolar free amino acids (Val, Ala, Iso, Leu, Gly, Met, Phe, Trp) in GL (solid boxes) and WL (striped boxes) of *P. zonale* plants prior to (5 d, grey) and after four (9 d) and eight days (13 d) of exposure to high light (HL, 800 µmol m^–2^ s^–1^, red) compared with those exposed to low light (LL, 180 µmol m^–2^ s^–1^, blue) under (**A**) optimal (25 °C, *HL*) and (**B**) lower (18 °C, *Cold + HL*) temperatures. Values are shown in µmol g^–1^ FW ± SE, *n* = 6–8. Different letters indicate statistically significant differences between different PAR intensities, time points, and leaf tissues (*p* < 0.05) according to Tukey’s post hoc test.

For almost all of the AAs measured, a significant difference in content between GL and WL in both experiments was observed (Figure 12 and Figure 13, significant “tissue” effects, Supplementary Table S6). This was particularly evident for Met, Asp, Val, Arg, Pro and Thr in the HL experiment and for Gly, Lys, Arg, Asn, Phe, Pro, Trp and Asp in the *Cold* + *HL* experiment. In GL, HL induced the increase in Ala, Leu, Thr and Glu at 25 °C, whereas at 18 °C it only stimulated increases in Thr and Gln (Figure 12 and Figure 13, significant “PAR” effects, Supplementary Table S6). However, in WL, HL provoked a significant increase in many other AAs: Gly, Val, Ile, Pro, Ser, Thr, Asp and Gln at 25 °C, while at 18 °C it caused an increase in Gly, Ile, Leu, Lys, Met, Phe, Asp and Trp and a decrease in Thr. The Gly/Ser ratio in the *Cold* + *HL* experiment was six times higher (*p* < 0.05) in the GL plants exposed to LL and up to 12 times higher (0.005 < *p* < 0.05) in the GL plants exposed to HL than in the corresponding treatment groups of the *HL* experiment.

In addition, the percentage of specific AAs in their total pool changed as a result of the different light and temperature intensities (Figure S2). Exposure to HL increased the Ala percentage and caused a decrease in the Asp fraction in GL at 25 °C. At the same time, the fraction of Tre, Gln and Ser in WL increased, while the percentages of Glu and Asn decreased with HL. As a result of higher EEE conditions (HL, lower temperature), the proportions of Ala and Tyr decreased, while the contributions of Thr and Gln in the total AA pool increased in GL. On the other hand, the proportions of Arg and Tyr in WL decreased significantly after 13 days under HL at 18 °C compared to the starting point of the *Cold* + *HL* experiment. Moreover, the Arg percentage in WL increased in the *Cold* + *HL* experiment compared to the *HL* experiment, while the Trp percentage in both tissue types was greater in *HL* compared to *Cold* + *HL*. The Arg percentage in WL was significantly higher than in GL at 18 °C. The lower temperature and HL increased the proportion of Thr and Pro in the total AA pool in both leaf tissue types compared to HL at 25 °C (Figure S2).

**Figure 14:**
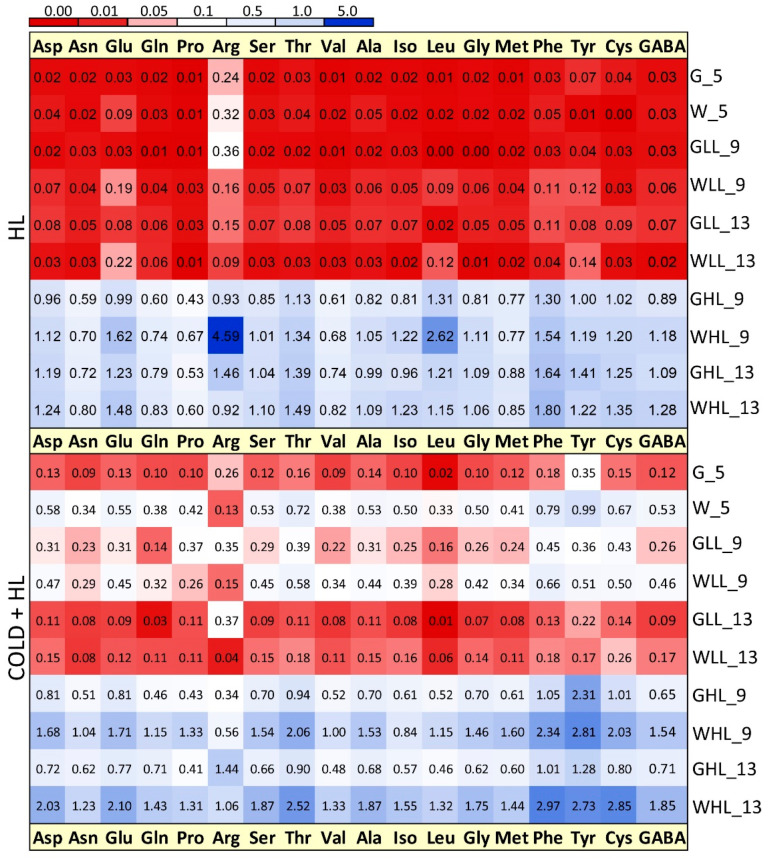
Distribution (%) of ^15^N free amino acids in GL and WL of *P. zonale* plants under optimal (*HL*) and lower temperatures (*Cold + HL*). The experimental conditions are explained in the legend to Figure 5. SE for each value was 2.1–19.7%; *n* = 4–5 and 1.1–17.9%; *n* = 3–7 in the *HL* and *Cold + HL* experiments, respectively.

The most notable effect of elevated EEE conditions (*Cold* + *HL* compared to the *HL* experiment) on certain AAs was the significant increase in Phe, Trp and Met content by almost 1.6-, 2.8-, and 2.1-fold, respectively, in WL (Figure 13 and Figure 14). The response of certain AAs in green leaf sectors to HL was more pronounced at 25 °C than at 18 °C (the increase in Ala, Leu and Glu in the *HL* experiment did not occur in the *Cold + HL* experiment, Figure 12 and Figure 13).

As for the distribution of AAs enriched in ^15^N, their increase under HL (both on day 9 and day 13) was most pronounced in both tissues and at both temperatures (Figure 14). It can be noted that the amount of ^15^N AA was generally higher in the *Cold* + *HL* experiment compared to *HL*. However, the highest content of all the ^15^N AAs among all the treatment groups was the content of the ^15^N-labelled Arg found in WL on the ninth day under HL in the *HL* experiment (Figure 14). Moreover, the highest levels of the ^15^N AAs in the *HL* experiment were calculated for Phe, Glu, Leu, Asp and Thr (all higher in WL under HL), while the ^15^N-labelled Gln, Pro, Val, Asn, and Met were less abundant. On the other hand, in the *Cold*+ *HL* experiment, a higher content of most of the measured ^15^N AAs was observed in WL than in GL. Among them, Phe, Tyr, Thr and Cys were the most abundant ^15^N AAs, especially on the last day of exposure to HL.

### 2.7. Gene Expression

**Figure 15:**
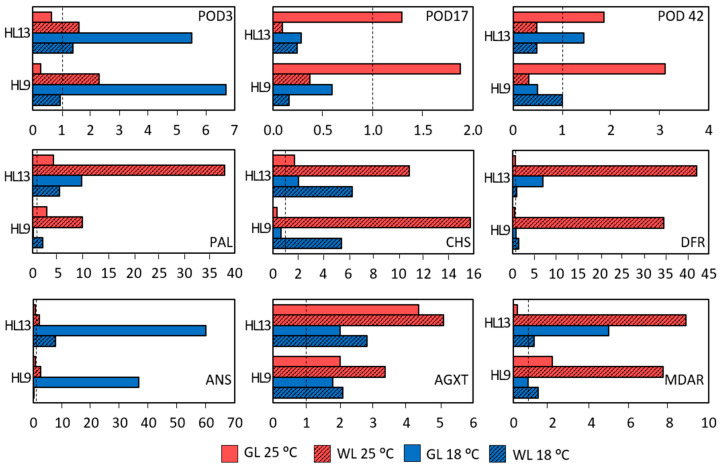
Relative expression of selected genes in GL (solid boxes) and WL (striped boxes) of *P. zonale* plants in comparison with the beginning (5 days) and after four (HL9) and eight days (HL13) of high light exposure (HL, 800 µmol m^–2^ s^–1^, red bars) at the optimal temperature of 25 °C (*HL*, red) and lower temperature of 18 °C (*Cold + HL*, blue). The values of the relative gene expression changes were calculated by applying the actin gene reference. *POD3*, *POD17*, *POD42*, *class III peroxidase isoforms*; *MDAR*, *monodehydroascorbate reductase*; *PAL*, *phenylalanine ammonia lyase*; *CHS*, *chalcone synthase*; *DFR*, *dihydroflavonol-4-reductase*; *ANS*, *anthocyanidin synthase*; *AGXT*, *alanine:glyoxylate aminotransferase*.

The transcript level of the gene encoding the specific peroxidase isoform POD3 was significantly induced (5–7 times) in GL during HL exposure at 18 °C, while the other isoform, POD42, was inducible in WL at 25 °C after four days of HL treatment (Figure 15). Gene encoding the alanine:glyoxylate aminotransferase (AGXT), a peroxisomal aminotransferase with a central role in photorespiration, was upregulated by almost 4–5 times in GL and WL under HL at 25 °C, and its upregulation was stronger in WL than in GL. A transient upregulation of gene encoding monodehydroascorbate reductase (MDAR), which is involved in the reduction of the monodehydroascorbate radical (MDA^•^) to dehydroascorbate (DHA) in the water–water and ascorbate–glutathione cycles, was observed in GL on the fourth day of HL exposure at 25 °C, while *MDAR* was even more strongly HL-induced in WL under the same conditions. (Figure 15). The light-inducible expression of key genes encoding the enzymes involved in the phenylpropanoid pathway, such as phenylalanine ammonia lyase (PAL, the first and crucial step), chalcone synthase (CHS, the first enzyme in flavonoid biosynthesis), and dihydroflavonol-4-reductase (DFR, a key enzyme in the reduction of dihydroflavonols to leucoanthocyanidins in both anthocyanin biosynthesis and proanthocyanidin accumulation) was generally more pronounced (10–40 times) in WL, particularly at the higher temperature (Figure 15). The most remarkable change in the gene expression induced by EEE conditions was observed for transcript encoding anthocyanidin synthase (ANS), the final enzyme of anthocyanidin biosynthesis, in GL at 18 °C.

## 3. Discussion

**Figure 16:**
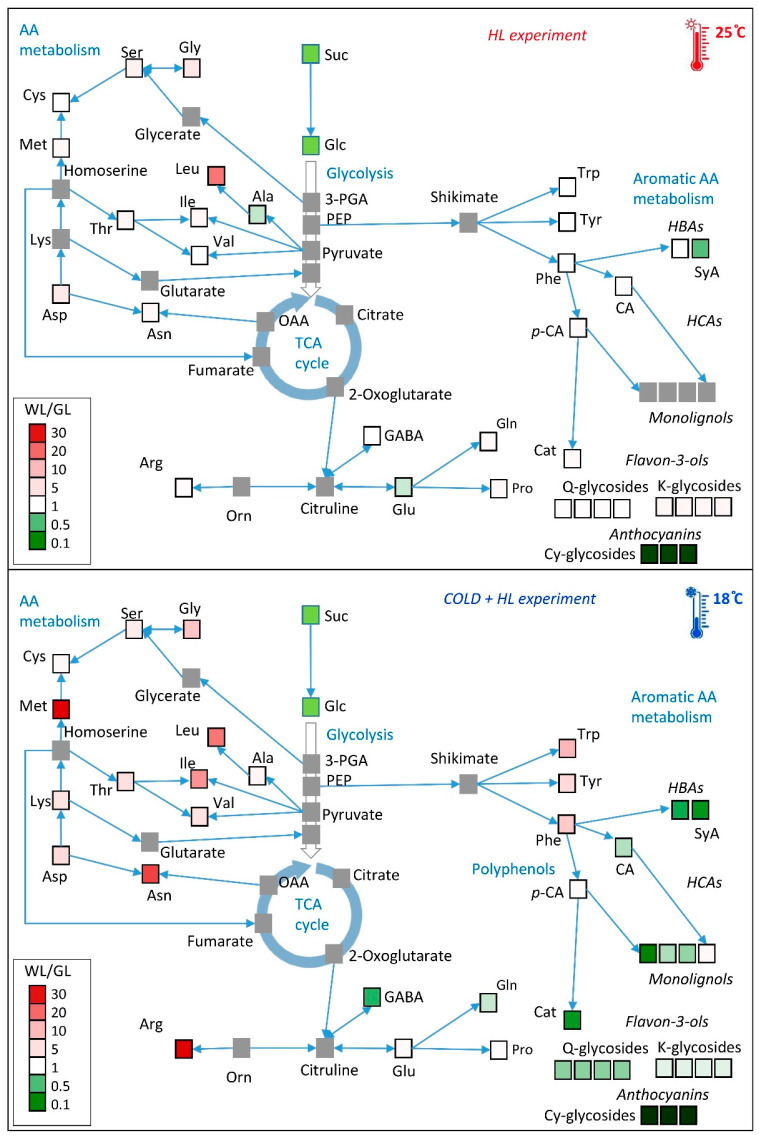
The main metabolic alterations of *P. zonale* GL and WL in response to treatments with HL and a lower temperature (18 °C). The colours indicate the WL/GL of each identified metabolite after eight days. Important metabolites that were not determined are marked in grey. CA, caffeic acid; Cat, catechin; Cy, cyanidin; GABA, γ-aminobutyric acid; Glc, glucose; HCA, hydroxycinnamic acids; K, kaempferol; OAA, oxaloacetate; *p*-HBA, *p*-hydroxybenzoic acid; *p*-CA, *p*-coumaric acid; PrcA, protocatechuic acid; Q, quercetin; Rha, rhamnose; Suc, sucrose; SyA, syringic acid; TCA, tricarboxylic acid cycle.

The results presented in this study show the effects of EEE on carbon and nitrogen allocation between the source - GL and sink - WL leaf tissues of variegated *P. zonale*. The EEE conditions were generated by a combination of high PAR intensity and lower temperature (18 °C), similar to Popova et al. [1]. We aimed to gain more insight into the benefits of constitutively higher levels of nitrogenous compounds and efficient antioxidants [19,21] in WL sectors for plant growth and development. The obtained responses of the measured metabolites of *P. zonale* WL and GL in the *HL* and the *Cold* + *HL* experiments are summarised in Figure 16.

### 3.1. Chlorophyll Content and Chlorophyll Fluorescence Parameters

Chlorophyll content is an important index for assessing photosynthetic capacity. It is assumed that the Chl content decreases under high light. In our study, the Chl content significantly decreased only in the last three days of the *HL* experiment at 25 °C under HL, while only a slight decrease was observed at 18 °C (Figure 2). In wheat, the Chl content showed no difference between plants grown at 250 and 500 µmol m^–2^ s^–1^ of PAR at 25 °C [25]. Chlorophyll content decreases under cold stress since lower temperatures reduce the activities of Chl biosynthetic enzymes [26]. During the greening process of rice seedlings, the Chl amount was significantly higher at 28 °C compared to 18 °C [27]. However, in our study, no effect of temperature on Chl content was found.

Although the Chl content was not significantly influenced by increased light intensity in GL, the ETR was strongly increased, similar to wheat [25]. The chlorophyll fluorescence parameters F_q_′/F_m_′ and *Ф*_PSII_ in *P. zonale* leaves did not differ significantly between the *HL* and *Cold + HL* experiments. The extent of the decrease in *F*_q_′/*F*_m_′ was higher in *Cold + HL*, indicating that PS II was more sensitive to HL at 18 °C than at 25 °C. This is not unexpected given that the electron pressure within the photosynthetic electron transport chain is higher at 18 °C than at 25 °C due to the activity reduction of the Calvin–Benson cycle enzymes.

The excitation energy at PS II can be dissipated via photosynthetic electron transport and directed into carbon fixation, or via the reduction of nitrite or other nutrients, and/or the water–water cycle [1]. The water–water cycle [28] refers to the photoreduction of O_2_ via a superoxide radical and H_2_O_2_ to water in PS I by electrons derived from water in PS II. The reduction of H_2_O_2_ occurs by Asc oxidation through the MDA^•^ to DHA by ascorbate peroxidase, MDAR, and dehydroascorbate reductase with a final consumption of reduced ferredoxin and/or NAD(P)H, allowing the safe dissipation of excess photon energy and electrons [29]. The transient *MDAR* upregulation on the fourth day of HL exposure at 25 °C and its strong upregulation after the eight-day HL exposure at 18 °C in GL (Figure 15) indicate the importance of the water–water cycle in EEE relaxation in GL. However, the 5–9-fold *MDAR* upregulation in WL in the *HL* experiment points to its role in H_2_O_2_ scavenging, and it could be related to the regulation of redox homeostasis in the cell wall, as well [30].

An additional EEE dissipation pathway is thermal dissipation by NPQ via the xanthophyll cycle [9]. The increase in NPQ from the onset of treatment with HL (Figure 1) indicated that the EEE in the PS II reaction centres can be relaxed by heat dissipation based on the xanthophyll cycle, allowing the protection of the PS II physiological function. This is evident from the *Ф*_PSII_ increase in *P. zonale* leaves at the end of both experiments (Figure 1). A similar recovery of PS II reaction centres by increased NPQ was observed in *Physocarpus opulifolius* after ten days of flooding stress [31]. Although NPQ depends on the violaxanthin de-epoxidase activity and de-epoxidation rate, which decreases at lower temperatures [32], no significant differences in NPQ were observed between 18 °C and 25 °C in our study (Figure 1). The results obtained, therefore, suggest that the influence of the transmembrane ΔpH under HL may be the predominant factor affecting NPQ. Since ATP synthase relies on ΔpH to produce ATP, the regulation of ATP synthase affects NPQ and electron transfer [33,34]. However, this assumption needs to be confirmed by measuring the amounts of xanthophylls, as well as the ATP/ADP and ATP/NADPH ratios.

### 3.2. Phenolics

Our results demonstrated an HL-induced accumulation of CA and *p*-CA in WL at 25 °C, while at 18 °C the accumulation of HBAs, Cat and Q in GL was more pronounced (Figure 4, Figure 5 and Figure 6). Kaempferol glycosides were more strongly induced by HL in WL than in GL at 25 °C. These changes were consistent with the increased levels of genes encoding PAL (the first and key step in the phenylpropanoid pathway) and CHS (the crucial enzyme in flavonoid biosynthesis), as seen in Figure 15. Moreover, these results fit very well with the increase in the level of EpFlav in both experiments, especially in *Cold* + *HL* (Figure 2). The upregulation of phenylpropanoid metabolism and the accumulation of polyphenols, especially anthocyanins, is considered crucial for the acclimation of plants to high light intensity and cold [35]. In our previous work, we demonstrated a strong stimulation of *ortho*-dihydroxy-B-ring-substituted phenylpropanoids (CA, Q, Cat and cyanidin, Cy) by high PAR intensity in GL of *P. zonale* plants [20]. On this basis, we proposed that the upregulated phenolics in GL have a more pronounced antioxidant role than the UV-B-shielding role of the specific phenolics induced in WL. Moreover, efficient sugar transport from the source-GL to the sink tissue - WL, stimulated by high light intensity, provided a building material for the biosynthesis of *p*-CA, K, and Q, mainly in the form of glycosides.

The possible function of flavonoids depending on their location in the leaf and their structural/antioxidant relationships (e.g., *ortho*-dihydroxy B-ring substitution) has already been discussed [12,36,37]. Higher levels of *ortho*-dihydroxy-B-ring-substituted flavonoids were found in the mesophyll cells of *Ligustrum vulgare* leaves in sunlight without UV radiation, which was considered to be part of the defence system against high-light-induced oxidative stress [36]. Nevertheless, the accumulated K and Q glycosides observed in the *HL* and *Cold* + *HL* experiments (Figure 6) have a higher capacity to inhibit ROS generation compared to other phenylpropanoids, and both can be synthesised in chloroplasts [11,14,38]. Similarly, the HL-induced accumulation of both *ortho*-dihydroxy-B-ring- and monohydroxy-B-ring-substituted flavonoids in coffee leaves has been reported [7]. In addition, phenolic compounds (flavonoids, anthocyanidins, and HCAs) serve as endogenous electron donors for vacuolar, apoplastic and cell-wall-bound PODs [15,16]. The oxidation of phenolics by H_2_O_2_, catalysed by PODs, implies the generation of phenoxyl radicals, which in turn are either polymerised or reduced by ascorbate (Asc) in the POXs/Phenolics/Asc H_2_O_2_-scavenging system [39].

Similar to our previous study [20], Cy glycosides were induced by HL exclusively in GL and their content was twice as high at 18 °C as at 25 °C (Figure 7 and Figure S1). This is consistent with the very strong (60-fold) upregulation of anthocyanidin synthase (ANS), the final enzyme of anthocyanidin biosynthesis, in GL at 18 °C (Figure 15). In addition, *DFR*, which is involved in the reduction of dihydroflavonols to leucoanthocyanidins in both anthocyanin biosynthesis and proanthocyanidin accumulation, was suppressed at 25 °C in GL but was seven times more strongly regulated at 18 °C. Proanthocyanidins (condensed tannins from catechin and epi-catechin) have not been identified in either GL or WL, so they may only act as precursors of anthocyanidins [40]. It has long been suspected that anthocyanins serve as photoprotectants in plants exposed to high irradiance. However, this is questionable as they absorb almost exclusively green photons, which are poorly absorbed by chlorophylls [41]. We suggest that the accumulation of Cy glycosides in the GL, as well as other polyphenols including the monolignols responsible for cell wall lignification, is an important energy escape valve and one of the main players in the consumption of reductants under EEE conditions. The synthesis of these flavonol glycosides is more expensive than that of HCAs and HBAs in terms of reduced carbon, reducing equivalents, and ATP costs [10]. The carbon diversion from primary metabolism to the expensive synthesis of secondary metabolites can account for 30% of the carbon flux and prevents downregulation of photosynthesis and photoinhibition [7]. Since this reprogramming of metabolism significantly increases the antioxidant capacity of GL, the HL-induced upregulation of flavonols could be an important strategy to divert excess energy into compounds with additional antioxidant activities [38].

### 3.3. Cell Wall Constituents

The plasticity of the cell wall is an important factor in the tolerance to HL. Cell walls are usually composed of structural proteins, cellulose, hemicellulose, pectin, and lignin, which increase their mechanical and water-resistance properties [42]. The experiments conducted here showed that the elevated EEE stimulates lignification and the induction of cellulose as the main component of the cell wall, as well as proteins in the cell wall of GL (Figure 9 and Figure 10). Under the same conditions, a higher level of pectin and hemicellulose (xyloglucans) was found in the cell wall of WL. The increase in HL-induced lignin at 18 °C in the GL cell wall was evidenced by a higher amount of its building block, *p*-coumaryl alcohol (Figure 11). This increase was also confirmed by non-invasive measurements (with Dualex 4 Scientific sensor), where the recorded signal came from HCAs in the cell wall in addition to EpFlav [43]. As previously mentioned, the POD-dependent polymerisation of polyphenols is closely related to cell wall lignification (reviewed in [16,44]). The HL induced a strong upregulation of the specific peroxidase isoform POD3 and POD17 at 18 °C and 25 °C, respectively, in GL. This could be correlated with the increase in HCA derivatives, monolignols, and lignin contents leading to cell wall stiffening (Figure 5, Figure 8, Figure 9, Figure 10, Figure 11 and Figure 15).

The EEE-induced increase in lignin and cellulose abundance in GL (leaf centre), as opposed to pectin and hemicellulose, served not only as an additional sink for reduced carbon, but also caused cell wall remodelling responsible for leaf thickening and curling to reduce light exposure. Similar morphological changes, such as leaf rolling due to changes in the contents of pectin and cell-wall-bound proteins, reduced reflectance and increased light transmittance in rice plants grown under HL [45]. In addition, plants grown in high-irradiance environments have thicker leaves and increased tissue lignification [46]. It has already been shown that lignin biosynthesis and monolignol content are stimulated by light [18,47].

### 3.4. Amino Acids

As reported in our previous study, the content of the majority of free AAs was higher in WL than in GL tissues of *P. zonale* plants grown under optimal conditions [19]. The content of AAs increased significantly in HL at both temperatures and in both leaf tissues (Table 1). In wheat and poplar leaves, the total pool of AAs also increased with increasing light intensity [25,48], although lower levels of several AAs were reported in HL-grown Arabidopsis [49]. It should be noted that both light intensity and light quality (red, blue, far red) affect the content and ratio of AAs in leaves and fruits [25,50,51].

The ^15^N-labelling experiment with K^15^NO_3_ allowed us to monitor the de novo synthesis of AAs in both tissue types. As the vascular bundles spread along the leaf margin, an additional ^15^NO_3_^–^ was available for both leaf sectors. Based on a recent differential transcriptome analysis between GL and WL published in Milić et al. [19], both leaf tissues are capable of synthesising all proteogenic amino acids de novo. Therefore, it is not possible to distinguish whether the ^15^N-labelled AAs in WL are the result of transport from GL or de novo synthesis in WL. Our study showed higher ^15^N enrichment in HL-treated plants in both tissue types and at both temperatures (Figure 14). In another experiment with variegated pelargonium leaf discs, the highest amount of ^15^N-labelled AAs was observed for Ala, Asp, Glu, and Ser and the lowest for Asn, Gly, Leu, Ile, Met, Tyr, and Val, regardless of the tissue considered [52]. The same study showed that 50% of the ^15^N atoms taken up by GL (increased by the presence of the white tissue) were actually exported to WL. In the *Cold* + *HL* experiment, the amount of ^15^N-labelled AAs was higher in WL than in GL, emphasising the role of AAs in a reprogrammed metabolic scheme under elevated EEE conditions (Figure 16).

The Glu-derived AA family (Arg, Pro, Glu, Gln) was the most abundant AA family in both tissues, with 30–46% in GL and 28–35% in WL (Supplementary Figure S2), similar to wheat leaves [25]. These AAs are of particular importance as they are associated with various stress responses. In our study, Pro, a hallmark of plant stress response, was not affected by EEE conditions in any of *P. zonale* leaf tissues (Figure 12). This response contrasts with the results found in HL-cultivated coffee plants where Pro was accumulated [7]. Besides its ROS scavenging properties, Pro is an important factor in cell wall fortification via hydroxyproline-rich glycoproteins or (hydroxyl)proline-rich proteins [53]. Its status in our study could be a consequence of its turnover, a balance between its synthesis and its involvement in HL-induced cell wall stiffening. Accordingly, an increased abundance of structural cell wall proteins was observed in WL at the end of the *Cold* + *HL* experiment (Figure 9 and Figure 12). As obtained in our study, the Pro content in wheat leaves was not altered by the change from 250 to 500 µmol m^–2^ s^–1^ of PAR at 25 °C [25].

Another Glu-derived AA, Arg, was significantly accumulated in WL at 18 °C compared to 25 °C, regardless of the light conditions (Figure 12, Figure 16 and Figure S2). The main product of ^15^N incorporation in WL was Arg, which showed a transient increase with a maximum after four days of exposure at 25 °C followed by an important decrease (Figure 14). Due to its high N/C ratio, Arg accumulates as a nitrogen storage compound that serves as a nitrogen transport compound, and its catabolism enables mobilisation between sink and source tissues [54]. This is consistent with the previously demonstrated remobilisation of nitrogen-rich compounds from WL to GL under N deficiency in variegated pelargonium [23]. On the other hand, Arg is a precursor of polyamines and nitric oxide (NO^•^), which is involved in the control of growth and the stress response [55]. The role of NO^•^ in the acclimation to EEE conditions has not been fully investigated. Distinguishing between ^15^NO^•^ and ^14^NO^•^ using electron paramagnetic resonance will allow an accurate determination of the source and biochemical pathways of NO^•^ in these two tissues in future studies. 

The percentage of GABA was increased in GL under HL at 18 °C (Figure 16). GABA is known to be a signalling molecule in plants, and its increase in GL may indicate its involvement in the EEE-dependent control of growth, development and stress acclimation [56].

As for the Ser-derived AA family, Gly and Ser contents were increased more by HL in WL than in GL, especially in the *Cold* + *HL* experiment (Figure 12, Figure 13 and Figure 16). These two AAs are directly involved in photorespiratory metabolism, which can only take place in GL of variegated *P. zonale* plants, as WL does not contain peroxisomes and has no catalase activity [20]. The Gly/Ser ratio, which is strongly correlated with an increased photorespiratory flux at high irradiances [57], was significantly increased by the lower temperature in GL of *P. zonale* plants in all treatment groups. The ammonia released from Gly during photorespiration is transferred back to Gly via Ser, Ala, or Asp. The 1.5-to-3-times-higher Ala content in GL of the *HL* compared to the *Cold* + *HL* experiment (Figure 13) confirms the negative correlation of Ala with photorespiratory flux proposed by Novitskaya et al. [57]. Moreover, the gene encoding peroxisomal AGXT, which plays a central role in photorespiration, was two- and four-fold upregulated at 18 °C and 25 °C, respectively, in GL (Figure 15). These results point to the importance of photorespiration in GL under HL as an additional alternative mechanism for the relaxation of EEE at PS II [9]. Despite the lack of a classic photorespiratory pathway in WL, this cell type has the ability to synthesise Gly by AGXT (the corresponding transcript is labelled as Cluster 20096.5336 and is more highly expressed in WL than in GL) [19]. Moreover, under EEE conditions, *AGXT* was upregulated 2–5 times in WL (more strongly than in GL) (Figure 15). Additionally, cells from WL can synthesise Ser de novo by serine hydroxymethyl transferase (the corresponding transcript is labelled as Cluster 20096.24970 and is more expressed in WL than in GL, [19]), which belongs to the glycolate pathway (photorespiration), and by 3-phosphoserine phosphatase, through the phosphorylated pathway [19]. In addition, Ser can be transferred from GL [52], as both tissue types harbour genes encoding various amino acid transporters [19]. As Ser plays an important role in regulating plant development and metabolism [58], it may play a role in mediating metabolic reprogramming in WL.

Besides Arg, Asn and Gln also serve as N storage and transport forms in many plant species due to their higher N/C ratio. The Gln/Glu ratio reflects the nitrogen status in plants [57,59]. In general, the Gln/Glu ratio was greater in WL, indicating a better nitrogen balance than in GL (except on the last day of the *Cold* + *HL* experiment). After a four-day exposure to HL in the *Cold + HL* experiment, a significantly higher Gln/Glu ratio was observed only in WL (0.005 < *p* < 0.05) compared with the *HL* experiment. Four days later, a higher Gln/Glu ratio was observed in GL (0.005 < *p* < 0.05) at 18 °C vs. 25 °C, and not in WL (Figure 12). These results indicate an improved nitrogen supply in GL on the last day of the *Cold* + *HL* experiment, possibly from the WL tissue. As mentioned above, Arg and other predominant AAs in WL were remobilised in GL in *P. zonale* plants facing nitrogen deficiency [23].

The branched-chain AAs (BCAAs) such as Val, Ile, and Leu were significantly increased in WL under elevated EEE conditions (Figure 13 and Figure 16). Moreover, the second major product of ^15^N incorporation in WL was Leu after four days at 25 °C (Figure 14). Regarding the biosynthetic pathway, the stimulation of the BCAA accumulation in WL represents a significant sink for reduced carbon. BCAAs, Pro, and Lys serve as alternative sources of respiratory substrates under stress conditions, as their oxidation via the tricarboxylic acid (TCA) cycle directly feeds electrons into the mitochondrial electron transport chain [54,60]. In addition, changes in BCAAs directly modulate TOR-related signals involved in the response to light [61,62]. TOR-dependent light signalling alters protein translation, cytoskeletal reorganisation, cell expansion, and proliferation [63].

A significantly higher content of the aromatic AAs, especially Phe, was only found in WL, especially at a lower temperature. Moreover, Phe and Tyr were the most abundant ^15^N-labelled AAs in the *Cold* + *HL* experiment in WL, although a high amount of ^15^N Tyr was also detected in GL after four days of HL exposure. This is significant from a biosynthetic perspective [64]. Downstream of chorismate, newly assimilated nitrogen was preferentially oriented to Phe and Tyr at the expense of Trp to provide precursors of hydroxycinnamates and monolignols, cell wall components, and the flavon-3-ols Q and K (K was higher in WL than in GL at 25 °C, Figure 6).

Our combined results demonstrate a clear restructuring of metabolism in both leaf tissues, enabling the variegated *P. zonale* plant to cope with EEE conditions. In short, the selected pools of almost all AAs, especially Arg and Met in WL and phenolic compounds in GL, were the main difference in these two leaf tissue types between the *HL* and *Cold* + *HL* experiments (Figure 16). In our previous work [20], we showed that HL (1350 µmol m^–2^ s^–1^ of PAR at 25 °C) triggered a five-fold increase in sucrose and a doubling of the glucose and fructose concentration in GL. In WL, the sucrose content was not altered, while the glucose and fructose concentrations doubled. Therefore, the high PAR intensity stimulated efficient sugar transport from the source to the sink leaf tissue and provided the building blocks for the synthesis of metabolites in WL, which are required for the EEE tolerance of the entire leaf. The majority of AAs that were largely accumulated by HL, especially at 18 °C in WL (Figure 16), are synthesised via energetically expensive pathways [65]. Therefore, we assume that HL-induced AA biosynthesis is part of a strategy to balance the redox status of the plant, which ultimately helps to avoid ROS generation and photo-oxidative damage.

## 4. Materials and Methods

### 4.1. Plant Material and Treatment

The model plant used in this experiment was the variegated *P. zonale*, cultivar “Frank Headley” [21]. Five weeks before the start of the experiment, the seedlings were vegetatively propagated by cuttings from the “mother plants” in small pots (6 × 6 × 5 cm) with Substrate 2 (Klasmann-Decilmann, Geeste, Germany) in the greenhouse.

**Figure 17:**
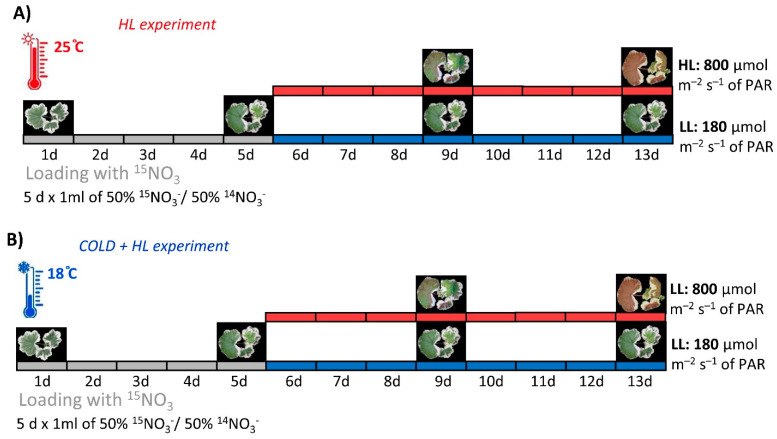
Schematic diagram of the experimental setup of the (**A**) *HL* experiment and (**B**) *Cold + HL* experiment. The photos of the plants represent the harvest times.

After one month, the plantlets were transferred to the growth chamber at 25/22 °C day/night temperature with a 16 h photoperiod (06:00–22:00 Central European Time, CET) and 180 µmol m^–2^ s^–1^ PAR. The experimental design was randomised, and the individual plants were rearranged daily in the chamber. Four to five randomly selected biological replicates were provided for each time point/treatment in each experiment and experimental replicate. In the *HL* experiment, plants were enriched with 3 mL of 15 mM KNO_3_ containing 98% of the isotope ^15^N for five days before half of the plants were exposed to high light intensity (HL, 800 µmol m^–2^ s^–1^ of PAR). The second half of the plants were exposed to low light (LL, 180 µmol m^–2^ s^–1^ of PAR) in the same chamber and under the same conditions as the first group (Figure 17A). In the *Cold + HL* experiment, the temperature was lowered to 18/16 °C (day/night) and the plants were enriched with ^15^N for five days. Similar to the *HL* experiment, half of the plants were exposed to HL (800 µmol m^–2^ s^–1^ of PAR), while the other half were exposed to LL (180 µmol m^–2^ s^–1^ of PAR) with the other conditions being the same (Figure 17B). At least three mature, fully light-exposed leaves were harvested from each plant at the beginning of the exposure (on the fifth day) and after four (on the 9th day) and eight days (on the 13th day). Sampling was performed in the middle of the photoperiod to exclude the effects of diurnal rhythms. Before storage at −80 °C for biochemical analysis, the green and white leaf sections were separated, weighed, pooled and frozen in liquid nitrogen. Three independent experiments were conducted for the two experimental setups *HL* and *Cold* + *HL*.

### 4.2. Morphological Leaf and Chlorophyll Fluorescence Parameters

The green and white leaf sections of light-exposed *P. zonale* leaves were determined after a six-day experiment and expressed as FW, while after drying (at 70 °C for 72 h) their DW was measured. Relative water content was measured with the smallest leaf discs (4 mm diameter) according to Sade et al. [66] to avoid sampling veins and possible overestimation effects.

All chlorophyll fluorescence measurements were performed daily between 09:00 and 10:00 (CET) on the green leaf sections. Chlorophyll fluorescence was measured with a miniPAM chlorophyll fluorimeter equipped with a light- and temperature-sensing leaf clip 2030-B (Heinz Walz GmbH, Effeltrich, Germany). The minimum fluorescence (F_0_) and the maximum fluorescence (F_m_) were measured in dark-adapted leaves in the early morning and the maximal photosynthetic efficiency of PS II (*Ф*_PSII_) was calculated as F_v_/F_m_ (F_v_ = F_m_ − F_0_), Fm was determined by applying a 1 s saturating flash of white light (4500 µmol m^−2^ s^−1^). Maximum fluorescence (F_m_′) and fluorescence (F) in light-adapted leaves were measured at midday, and the operating PS II efficiency, F_q_′/F_m_′ = (F_m_′ − F)/F_m_′, was estimated according to Baker [67]. NPQ was calculated according to the Stern–Volmer equation NPQ = (F_m_ − F_m_′)/F_m_′ [32]. The fluorescence parameters of the individual plant were mean values of three leaves.

### 4.3. Dynamics of Epidermal Flavonoids and Total Chlorophyll Accumulation

The in vivo contents of total Chl and EpFlav contents were measured daily, three to four hours after light incidence using the Dualex 4 Scientific sensor (FORCE-A, Orsay, France; see Cerović et al. [43] for more details). All measurements were carried out on a selected, mature, healthy leaf within the green sector.

### 4.4. Analysis of the Polyphenols

The frozen WL and GL material was rapidly homogenised in liquid nitrogen and extracted in methanol containing 0.1% HCl, followed by acid hydrolysis to determine aglycones, as previously described in [20]. The extractions were carried out in duplicate and subsequently purged with nitrogen.

Analyses were performed by HPLC coupled to a photodiode array detector (Ultimate 3000, Thermo Fisher Scientific, Waltham, MA, USA) on a 250 × 4.6 mm, 5.0 mm, Luna C18 (2) reversed phase column (Phenomenex Ltd. Torrance, CA, USA). The elution conditions were exactly as previously described [19]. Specific phenolic compounds were identified by comparing the absorption spectra with authentic standards and by spiking. Quantification was based on peak area using Chromeleon CDS 6.8 (Thermo Fisher Scientific, USA).

### 4.5. Cell Wall Isolation and Purification

The plant cell wall was isolated following the procedure described by Vidović et al. [24]. Series of extractions with organic solvents were performed to remove pigments, alkaloids, tannins, soluble sugars and other low-molecular-weight metabolites from the cell wall residues. Leaf sectors were pulverised with a mortar and pestle in liquid nitrogen and extracted in 80% methanol (1/8, *w*/*v*) while shaking for 60 min at room temperature. The homogenate was centrifuged at 1000× *g* for 20 min at room temperature and the pellet was washed twice with 80% methanol. The pellet was resuspended in 1 M NaCl containing 0.5% Triton X-100 and centrifuged at 1000× *g* for 20 min at room temperature. The pellet was rinsed with distilled water, once with absolute methanol and twice with acetone. The purified cell wall was dried and used for structural analyses.

### 4.6. Analysis of Cell-Wall-Bound Phenolics

Cell-wall-bound phenolics were isolated from the obtained cell wall material by alkaline hydrolysis in 1 M warm (80 °C) NaOH over 17 h at room temperature [68]. The HPLC method described above was used to determine free phenolic compounds in neutralised hydrolysates.

### 4.7. Infrared (IR) Spectroscopy of the Cell Wall Samples

FTIR was used to profile the WL and GL cell walls. The FTIR spectra of the extracted cell wall materials were recorded using a Perkin Elmer Spectrum Two equipped with the Universal ATR accessory. The spectrum of each powder sample was recorded in the range 4000–400 cm^−1^ with 200 scans and a spectral resolution of 4 cm^−1^. The spectral data obtained show the superposition of spectral signatures of the carbohydrates, proteins, and phenolic cell wall polymers, and the identification of the different chemical functional groups was performed by comparison with reference data [24,69]. Baseline correction was performed using Spectra Gryph software (https://www.effemm2.de/spectragryph/index.html, accessed 7 December 2022).

### 4.8. Amino Acid Analysis and the Enrichment of Amino Acids with ^15^N

Leaf tissues (approximately 100 mg) were homogenised into a fine powder with liquid nitrogen using steel beads (3 mm) and Qiagen’s TissueLyser II vibratory mill at a frequency of 30 Hz for 100 s. The obtained powder was mixed with 800 µL of 50% methanol and 800 µL of chloroform, shaken for 1 h at 4 °C in the dark and centrifuged for 10 min at 16,000× *g* at 4 °C. The upper methanol phase was removed, separated, and dried in a speed-vac for amino acid analysis [19].

The AA content was determined by HPLC after derivatisation following the method described in Milić et al. [19]. AA derivatives were identified and quantified using HPLC–PDA (LC-20AB Prominence Liquid Chromatograph, Shimadzu, Kyoto, Japan) with fluorescence detection. The excitation wavelength was set to 340 nm and the emission wavelength to 450 nm. The elution gradient was set using 20 mM sodium phosphate buffer pH 6.8: methanol: tetrahydrofuran (THF, 90:9:1, *v*:*v*:*v*) and 20 mM sodium phosphate buffer pH 6.8: methanol: THF (40:59:1, *v*:*v*:*v*) following Milić et al. [19]. The injection volume was 30 µL.

The Pro content was measured spectrophotometrically according to Milić et al. [19]. The frozen leaf tissue was homogenised in liquid nitrogen, extracted in 3% sulphosalicylic acid (1:10, *w*:*v*), mixed with ninhydrin and glacial acetic acid, and incubated at 100 °C for 60 min. The reaction was stopped in an ice bath and toluene was added. The upper organic phase was used to measure the absorbance at 520 nm. Pro content was determined using a standard curve.

Gas chromatography coupled with mass spectrometry (GC–MS) was used to measure the AAs enriched in ^15^N [70]. The extracted AAs were purified by ion exchange chromatography using Dowex 50WX8 resin as described in Cukier et al. [71]. A standard (2 μL, 2.5 mM α-aminobutyric acid in deionised water) was added as a quantification standard to a known volume of the AA extract and the mixture was reduced to dryness in sealable microtubes under vacuum. A freshly prepared derivatisation consisting of 15:15:1 (*v*/*v*/*v*) N-methyl-N-tert-butyldimethylsilyl-trifluoroacetamide (MBDSTFA)/acetonitrile (ACN)/Tris, acetate, and ethylenediaminetetraacetic acid buffer (TEA) were added to the dried extract and the microtubes were thoroughly shaken, centrifuged and incubated in a heating block (95 °C, 30 min) [71]. After cooling, 25 μL of the derivatised extracts were injected (0.25 μL, split mode-4) into the GC–MS system containing a BR5MS column (5% diphenyl/95% dimethylpolysiloxane, 30 m × 0.25 mm i.d., 0.25 μm film thickness, Bruker, Germany) mounted in a 436-GC coupled to a single quadruple (SQ)SCION MS (Bruker, Germany). The GC was operated in constant pressure mode with helium as the carrier gas (initial flow, 1 mL min^−1^). The temperatures of the injector, MS transfer line and source were 280 °C, 290 °C, and 220 °C, respectively. The chromatogram was developed with a thermal gradient of 60 °C for 1 min, a linear gradient of 30 °C min^−1^ to 120 °C, a linear gradient of 8 °C min^−1^ to 300 °C, followed by 300 °C for 5 min, resulting in a total run of 39 min [71]. The MS was programmed in single-ion monitoring (SIM) mode, with a mass range m/z of 50–650 Da, an electron impact (EI) ionisation of 70 eV, and we monitored the mass (M) of the largest significant component, mass plus 1 (M + 1) and mass plus 2 (M + 2) ions for each AA. A separation of 22 AAs was achieved, and quantification was carried out using calibration curves [70]. The enrichment of each AA with ^15^N (derived from the labelling) was calculated according to [71].

### 4.9. qPCR

Plant tissue was frozen in liquid nitrogen and ground using a mortar and pestle. Total RNA was extracted from 300 mg of WL and GL following the cetrimonium bromide (CTAB)-based protocol described in Vidović and Ćuković [72]. To remove any remaining DNA prior to cDNA synthesis, total RNA samples were treated with the Ambion^®^ DNA-free™ DNase Treatment and Removal DNA kit. cDNA synthesis was performed according to the Thermo Fisher Scientific protocol using Random Hexamer Primer and the RevertAid™ Reverse Transcriptase. 

Prior to the SYBR Green assay, total cDNAs were diluted 1:4 with nuclease-free water. Reactions were performed in a volume of 25 µL containing 300 nM of each primer and 1X SYBR Green PCR Master Mix (Thermo Scientific). Real-time PCR was conducted on the Mic Real Time PCR Cycler (Bio Molecular Systems, Brisbane, Queensland, Australia) under the following cycles: 2 min at 50 °C, 10 min at 95 °C and 40 cycles of (95 °C for 15 s, 60 °C for 1 min). Each PCR reaction was performed in duplicate and no-template controls were included. Amplification of PCR products was detected in real time and results were analysed with micPCR software (Bio Molecular Systems, Brisbane, Queensland, Australia) and presented as 2^-dCt^. Primers used for gene expression analysis were designed using Primer 3 software based on our previously performed de novo transcriptome sequencing recently published in Milić et al. [19]. Primer pairs (Supplementary Table S7) included the genes related to antioxidant metabolism: *class III peroxidase isoforms POD3*, *POD17*, *POD42*; *monodehydroascorbate reductase*, *MDAR*; *phenylpropanoid metabolism: phenylalanine ammonia lyase*, *PAL*; *chalcone synthase*, *CHS; dihydroflavonol-4-reductase*, *DFR*; *anthocyanidin synthase*, *ANS*; and amino acid metabolism: *alanine:glyoxylate aminotransferase*, *AGXT.* The values of the relative gene expression changes were calculated by applying the actin gene reference.

### 4.10. Statistics

The biochemical data from three independent experiments were evaluated and the standard deviations are given in the figures. After checking the prerequisites, the three-way ANOVA without replication was used to show the effects of light intensity, time, tissue type and their interactions on the content of phenolic compounds, amino acids and FW/DW ratio in the green and white leaf parts of *P. zonale* plants for each functional group. Homogeneity of variance was checked with Levene’s test. Tukey’s post hoc test was used to test for significant differences in phenolics, amino acids and FW/DW ratios between the different treatment groups. The threshold for significance was set at 0.05.

One-way repeated-measures ANOVA was used to test for differences in photosynthetic parameters, EpFlav, and Chl accumulation during the experiment (within-subject factor) in plants exposed to different light regimes (between-subject factors). To test for significant differences in phenolic compounds, EpFlav, Chl content, and Chl fluorescence parameters between LL and HL, the Mann–Whitney U/*t*-test was used and the significance threshold value was set at 0.05.

ANOVA, Mann–Whitney U-test and Tukey’s post hoc test were performed using the statistical software IBM SPSS (version 20.0, SPSS Inc., Chicago, IL, USA).

## 5. Conclusions

To balance energy supply with photosynthetic capacity and avoid photo-oxidative damage, plants have evolved various mechanisms for energy dissipation and protection from high light. The present work provides evidence that the WL of the variegated leaf acts as an important energy escape valve required to protect GL under the EEE conditions. Our results show that both primary and secondary metabolism in these two contrasting leaf tissues are extensively reprogrammed under high light irradiation. This included the accumulation of a number of free AAs (predominantly in WL) and flavonoid glycosides K, Q (in WL and GL). At lower temperatures, HL exposure led to an even greater accumulation of AAs (namely Arg, Asn, branched-chain and aromatic amino acids) in WL and to an accumulation of Cy glycosides and cell wall stiffening in GL. These metabolic changes were primarily related to the provision of an additional energy escape valve to maintain the photosynthetic performance of GL under EEE conditions. Our results not only provide additional evidence for the benefits of variegated leaves as a model system, but also enable a better understanding of the adaptive value of variegation in the absence of consistent conclusions on its ecological benefits [52]. Finally, our study paves the way to a better understanding of responses to challenging environmental conditions, such as low temperatures and high light intensities, that affect photosynthesis and yield in plants and crops.

## Figures and Tables

**Table 1:** Total free amino acid (AA) content in GL and WL of *P. zonale* plants at the beginning (5 d) and after four (9 d) and eight days (13 d) of high light exposure (HL, 800 µmol m^–2^ s^–1^) compared to those exposed to low light (LL, 180 µmol m^–2^ s^–1^, blue bars) under optimal (25 °C, *HL*) and lower (18 °C, *Cold + HL*) temperatures.

	G_5	GLL_9	GLL_13	GHL_9	GHL_13	W_5	WLL_9	WLL_13	WHL_9	WHL_13
*HL*	3.6 ± 0.3 ^a^	4.8 ± 0.5 ^ab^	5.4 ± 0.2 ^abc^	6.5 ± 0.2 ^bcde^	7.4 ± 0.4 ^cdef^	8.6 ± 0.6 ^ef^	6.2 ± 0.3 ^bcd^	9.6 ± 0.4 ^fg^	7.8 ± 0.9 ^def^	11.4 ± 0.9 ^g^
*Cold + HL*	5.1 ± 0.4 ^a^	6.9 ± 0.3 ^a^	7.3 ± 0.7 ^a^	5.8 ± 0.7 ^a^	7.3 ± 0.7 ^a^	17.3 ± 1.8 ^b^	15.6 ± 0.6 ^b^	16.1 ± 1.1 ^b^	13.1 ± 0.7 ^b^	22.4 ± 2.1 ^c^

Values are given in µmol g^–1^ FW ± SE, *n* = 6–8. Different letters indicate statistically significant differences between different PAR intensities and different leaf tissues (*p* < 0.05).

## Data Availability

The data presented in this study are available in Supplementary Materials, that can be downloaded at: https://www.mdpi.com/article/10.3390/ijms24032269/s1.

## Supplementary Materials

The following supporting information can be downloaded at: https://www.mdpi.com/article/10.3390/ijms24032269/s1.
