# Supplementary material for: Carbon and Nitrogen Allocation between the Sink and Source Leaf Tissue in Response to the Excess Excitation Energy Conditions
Source: Int J Mol Sci. 2023 Jan 23;24(3):2269. doi: 10.3390/ijms24032269 (PMC9917124; doi:10.3390/ijms24032269)
Supplement: Supplementary file 1 [file ijms-24-02269-s001.zip › Table S1.pdf]

Table S1. Repeated-measures analysis of variance (profile analysis) on photosynthetic parameters determined during 13 days of the *HL* and *Cold+HL* experiments in the leaves of *P. zonale* plants. Experimental conditions were as described in Figure 1. The *Df*s are shown in the brackets (the first number represents the *Df* of main effects and their interactions and the second number is the *Df* of error). Other abbreviations are explained in Figure 1.

HL experiment, *source*								
Between-subjects	F_q'/F_m' *(Df: 1; 18)*		Φ_{PSII} *(Df: 1; 18)*		ETR *(Df: 1; 18)*		NPQ *(Df: 1; 18)*	
	F	*P > F*	*F*	*P > F*	*F*	*P > F*	*F*	*P > F*
PAR	**114.33**	**< 0.0001**	**11.97**	**0.0030**	**643.11**	**< 0.0001**	**354.64**	**< 0.0001**
Within-subjects	F_q'/F_m' *(Df: 1; 12)*		Φ_{PSII} *(Df: 1; 12)*		ETR *(Df: 1; 12)*		NPQ *(Df: 1; 12)*	
Day	**5.81**	**< 0.0001**	1.56	0.2237	**34.46**	**< 0.0001**	**16.78**	**< 0.0001**
Day × PAR	**6.78**	**< 0.0001**	2.01	0.1570	**30.42**	**< 0.0001**	**14.54**	**< 0.0001**

COLD + HL experiment*, source*								
Between-subjects	F_q'/F_m' *(Df: 1; 42)*		Φ_{PSII} *(Df: 1; 42)*		ETR *(Df: 1; 42)*		NPQ *(Df: 1; 42)*	
	F	*P > F*	*F*	*P > F*	*F*	*P > F*	*F*	*P > F*
PAR	**340.75**	**< 0.0001**	55.30	0.5684	**698.46**	**< 0.0001**	299.0	0.8768
Within-subjects	F_q'/F_m' *(Df: 1; 12)*		Φ_{PSII} *(Df: 1; 12)*		ETR *(Df: 1; 12)*		NPQ *(Df: 1; 12)*	
Day	**34.80**	**< 0.0001**	**7.64**	**< 0.0001**	**111.01**	**< 0.0001**	**57.42**	**< 0.0001**
Day × PAR	**32.26**	**< 0.0001**	**8.25**	**< 0.0001**	**103.93**	**< 0.0001**	**52.16**	**< 0.0001**
