# Supplementary material for: Carbon and Nitrogen Allocation between the Sink and Source Leaf Tissue in Response to the Excess Excitation Energy Conditions
Source: Int J Mol Sci. 2023 Jan 23;24(3):2269. doi: 10.3390/ijms24032269 (PMC9917124; doi:10.3390/ijms24032269)
Supplement: Supplementary file 1 [file ijms-24-02269-s001.zip › Table S2.pdf]

**Table S2.** Repeated-measures analysis of variance (profile analysis) on EpFlav and Chl accumulation determined during 13 days of the *HL* and *Cold+HL* experiments in the leaves of *P. zonale* plants. Experimental conditions were as described in Figure 2. The *Df*s are shown in the brackets (the first number represents the *Df* of main effects and their interactions and the second number is the *Df* of error). Other abbreviations are explained in Figure 2.

| ***HL experiment,*** *source* | | | | |
|---|---|---|---|---|
| *Between-subjects* | EpFlav *(Df: 1; 24)* | | Chl *(Df: 1; 24)* | |
| | *F* | *P > F* | *F* | *P > F* |
| PAR | **97.23** | **< 0.0001** | **13.50** | **0.0012** |
| *Within-subjects* | EpFlav *(Df: 1; 12)* | | Chl *(Df: 1; 12)* | |
| Day | **194.50** | **< 0.0001** | **2.33** | **0.0474** |
| Day × PAR | **148.05** | **< 0.0001** | 1.55 | 0.1798 |
| ***COLD + HL experiment****, source* | | | | |
| *Between-subjects* | EpFlav *(Df: 1; 24)* | | Chl *(Df: 1; 24)* | |
| | *F* | *P > F* | *F* | *P > F* |
| PAR | **315.82** | **< 0.0001** | 0.02 | 0.8837 |
| *Within-subjects* | EpFlav *(Df: 1; 12)* | | Chl *(Df: 1; 12)* | |
| Day | **249.86** | **< 0.0001** | **2.3874** | **0.0300** |
| Day × PAR | **208.86** | **< 0.0001** | 1.1568 | 0.3325 |
