# Supplementary material for: Carbon and Nitrogen Allocation between the Sink and Source Leaf Tissue in Response to the Excess Excitation Energy Conditions
Source: Int J Mol Sci. 2023 Jan 23;24(3):2269. doi: 10.3390/ijms24032269 (PMC9917124; doi:10.3390/ijms24032269)
Supplement: Supplementary file 1 [file ijms-24-02269-s001.zip › Table S3.pdf]

Table S3. Three-way ANOVA results for the effects of tissue type (GL and WL), time point (9th and 13th day) and two PAR intensities as well as their interaction on the content of phenolic compounds found in the leaves of *P. zonale* plants. Experimental conditions were as described in Figure 4. The *Df*s are shown in the brackets (the first number represents *Df* of main effects and their interactions and the second number is *Df* of error). CA, caffeic Cat: catechin, acid, HBAs, total hydroxybenzoic acids; K, kaempferol; *p*-CA, *p*-coumaric acid; *p*-CA der, *p*-coumaric acid derivative; Q, quercetin; SyA, syringic acid.

**HL experiment**							
Trait	Source of variation	*F*	*P* > *F*	Trait	Source of variation	*F*	*P* > *F*
**Cat**	**tissue**	**10.51**	**0.0020**	**SyA**	**tissue**	**102.60**	**< 0.0001**
(*Df*: 1; 59)	**time**	**10.85**	**< 0.0001**	(*Df*: 1; 59)	**time**	**7.05**	**0.0020**
	PAR	0.06	0.8010		**PAR**	**62.07**	**< 0.0001**
	tissue × time	2.94	0.0620		tissue × time	0.44	0.6480
	tissue × PAR	1.88	0.1760		**tissue × PAR**	**10.96**	**0.0020**
	time × PAR	0.11	0.7420		time × PAR	0.04	0.8480
	**tissue × time × PAR**	**7.45**	**0.0090**		**tissue × time × PAR**	**6.70**	**0.0130**
**HBAs**	**tissue**	**6.38**	**0.0150**	**CA**	**tissue**	**5.43**	**0.0240**
(*Df*: 1; 59)	**time**	**13.14**	**< 0.0001**	(*Df*: 1; 59)	**time**	**13.41**	**< 0.0001**
	PAR	1.87	0.1780		**PAR**	**70.68**	**< 0.0001**
	**tissue × time**	**6.00**	**0.0050**		tissue × time	1.75	0.1840
	tissue × PAR	0.17	0.6850		tissue × PAR	0.15	0.7010
	time × PAR	0.08	0.7810		**time × PAR**	**33.86**	**< 0.0001**
	**tissue × time × PAR**	**20.20**	**< 0.0001**		tissue × time × PAR	2.65	0.1100
***p*-CA**	**tissue**	**29.54**	**< 0.0001**	***p*-CA der**	**tissue**	**26.7**	**< 0.0001**
(*Df*: 1; 59)	**time**	**4.32**	**0.0190**	(*Df*: 1; 59)	time	3.0	0.0590
	**PAR**	**25.32**	**< 0.0001**		**PAR**	**31.1**	**< 0.0001**
	tissue × time	0.52	0.5980		tissue × time	0.1	0.9360
	tissue × PAR	1.65	0.2050		tissue × PAR	3.2	0.0770
	time × PAR	2.84	0.0980		time × PAR	1.3	0.2690
	tissue × time × PAR	0.52	0.4730		tissue × time × PAR	0.1	0.7140
**Q**	**tissue**	**44.08**	**< 0.0001**	**K**	**tissue**	**59.04**	**< 0.0001**
(*Df*: 1; 59)	**time**	**12.81**	**< 0.0001**	(*Df*: 1; 59)	**time**	**18.19**	**< 0.0001**
	PAR	1.97	0.1670		**PAR**	**51.01**	**< 0.0001**
	tissue × time	2.10	0.1330		tissue × time	3.05	0.0560
	**tissue × PAR**	**5.59**	**0.0220**		**tissue × PAR**	**14.34**	**< 0.0001**
	time × PAR	0.18	0.6750		**time × PAR**	**4.50**	**0.0390**
	tissue × time × PAR	0.81	0.3730		tissue × time × PAR	0.83	0.3660
**COLD + HL experiment**							
Trait	Source of variation	*F*	*P* > *F*	Trait	Source of variation	*F*	*P* > *F*
**Cat**	**tissue**	66.76	**< 0.0001**	**SyA**	**tissue**	**235.14**	**< 0.0001**
(*Df*: 1; 69)	**time**	24.60	**< 0.0001**	(*Df*: 1; 59)	**time**	**15.46**	**< 0.0001**
	**PAR**	41.31	**< 0.0001**		**PAR**	**133.39**	**< 0.0001**
	**tissue × time**	25.88	**< 0.0001**		**tissue × time**	**12.61**	**< 0.0001**
	**tissue × PAR**	44.37	**< 0.0001**		**tissue × PAR**	**64.19**	**< 0.0001**

	**time × PAR**	69.09	**< 0.0001**		**time × PAR**	**21.18**	**< 0.0001**
	**tissue × time × PAR**	33.17	**< 0.0001**		**tissue × time × PAR**	**7.86**	**0.0070**
**HBA**	**tissue**	**70.83**	**< 0.0001**	**CA**	tissue	0.02	0.8840
(*Df*: 1; 69)	**time**	**6.11**	**0.0040**	(*Df*: 1; 69)	time	1.84	0.1670
	**PAR**	**28.10**	**< 0.0001**		**PAR**	**11.39**	**0.0010**
	**tissue × time**	**3.81**	**0.0280**		**tissue × time**	**3.73**	**0.0300**
	**tissue × PAR**	**12.17**	**0.0010**		tissue × PAR	0.12	0.7360
	**time × PAR**	**17.88**	**< 0.0001**		time × PAR	1.95	0.1680
	tissue × time × PAR	2.05	0.1570		tissue × time × PAR	0.49	0.4860
***p*-CA**	**tissue**	**57.16**	**< 0.0001**	***p*-CA der**	**tissue**	**64.35**	**< 0.0001**
(*Df*: 1; 69)	**time**	**19.99**	**< 0.0001**	(*Df*: 1; 69)	**time**	**20.11**	**< 0.0001**
	PAR	0.65	0.4230		PAR	1.04	0.3130
	tissue × time	1.77	0.1790		**tissue × time**	**3.16**	**0.0490**
	tissue × PAR	0.03	0.8620		tissue × PAR	0.28	0.6010
	**time × PAR**	**9.16**	**0.0040**		time × PAR	3.16	0.0810
	tissue × time × PAR	2.35	0.1300		tissue × time × PAR	1.13	0.2930
**Q**	tissue	0.43	0.5170	**K**	tissue	1.04	0.3110
(*Df*: 1; 59)	**time**	**9.90**	**< 0.0001**	(*Df*: 1; 69)	**time**	**58.31**	**< 0.0001**
	**PAR**	**114.92**	**< 0.0001**		**PAR**	**158.79**	**< 0.0001**
	tissue × time	0.81	0.4490		tissue × time	0.17	0.8430
	**tissue × PAR**	**15.54**	**< 0.0001**		**tissue × PAR**	**4.49**	**0.0380**
	**time × PAR**	**34.08**	**< 0.0001**		**time × PAR**	**49.14**	**< 0.0001**
	tissue × time × PAR	0.29	0.5950		tissue × time × PAR	0.00	0.9950
