# Supplementary material for: Carbon and Nitrogen Allocation between the Sink and Source Leaf Tissue in Response to the Excess Excitation Energy Conditions
Source: Int J Mol Sci. 2023 Jan 23;24(3):2269. doi: 10.3390/ijms24032269 (PMC9917124; doi:10.3390/ijms24032269)
Supplement: Supplementary file 1 [file ijms-24-02269-s001.zip › Table S4.pdf]

Table S4. Two-way ANOVA results for the effects of time point (9th and 13th day) and two PAR intensities as well as their interaction on the Fw/Dw ratio *P. zonale* leaves. Experimental conditions were as described in Figure 4. The *Df*s are shown in the brackets (the first number represents the *Df* of the main effects and their interactions and the second number is *Df* of error).

Trait	Source of variation	*F*	*P* > *F*	Trait	Source of variation	*F*	*P* > *F*
FW/DW	**time**	**12.74**	**0.0001**	FW/Dw	Time	1.23	0.3054
HL	**PAR**	**21.25**	**0.0001**	*COLD+HL*	**PAR**	**35.06**	**< 0.0001**
(*Df*: 1; 39)	time × PAR	0.05	0.8217	(*Df*: 1; 39)	time × PAR	1.52	0.2257
