# Supplementary material for: Carbon and Nitrogen Allocation between the Sink and Source Leaf Tissue in Response to the Excess Excitation Energy Conditions
Source: Int J Mol Sci. 2023 Jan 23;24(3):2269. doi: 10.3390/ijms24032269 (PMC9917124; doi:10.3390/ijms24032269)
Supplement: Supplementary file 1 [file ijms-24-02269-s001.zip › Table S5.pdf]

Table S5. Three-way ANOVA results for the effects of tissue type (GL and WL), time point (9th and 13th day) and two PAR intensities as well as their interaction on the content of the cell-wall bound phenolic compounds found in the leaves of *P. zonale* plants. Experimental conditions were as described in Figure 4. The *Df*s are shown in the brackets (the first number represents *Df* of the main effects and their interactions and the second number is *Df* of error). CA der, caffeic acid derivative; *p*-CA, *p*-coumaric acid.

**COLD + HL experiment**							
Trait	Source of variation	*F*	*P* > *F*	Trait	Source of variation	*F*	*P* > *F*
***p*-CA**	tissue	2.49	0.1199	**CA der 2**	**tissue**	**65.55**	**< 0.0001**
**aldehyde**	**time**	**6.58**	**0.0026**	(*Df*: 1; 69)	**time**	**39.58**	**< 0.0001**
(*Df*: 1; 69)	**PAR**	**20.23**	**< 0.0001**		**PAR**	**53.52**	**< 0.0001**
	tissue × time	2.17	0.1231		**tissue × time**	**34.18**	**< 0.0001**
	tissue × PAR	3.06	0.0853		**tissue × PAR**	**51.83**	**< 0.0001**
	**time × PAR**	**13.00**	**0.0006**		**time × PAR**	**50.44**	**< 0.0001**
	**tissue × time × PAR**	**10.21**	**0.0022**		**tissue × time × PAR**	**51.11**	**< 0.0001**
***p*-CA**	**tissue**	**48.58**	**< 0.0001**	***p*-CA**	**tissue**	**10.06**	**0.0024**
(*Df*: 1; 69)	time	3.11	0.0519	**alcohol**	**time**	**6.35**	**0.0031**
	**PAR**	**11.88**	**0.0010**		**PAR**	**19.65**	**< 0.0001**
	tissue × time	1.25	0.2943	(*Df*: 1; 69)	tissue × time	0.06	0.9450
	tissue × PAR	0.00	0.9650		**tissue × PAR**	**16.66**	**0.0001**
	**time × PAR**	**6.36**	**0.0144**		**time × PAR**	**4.83**	**0.0319**
	tissue × time × PAR	1.52	0.2223		tissue × time × PAR	3.57	0.0637
