# Supplementary material for: Carbon and Nitrogen Allocation between the Sink and Source Leaf Tissue in Response to the Excess Excitation Energy Conditions
Source: Int J Mol Sci. 2023 Jan 23;24(3):2269. doi: 10.3390/ijms24032269 (PMC9917124; doi:10.3390/ijms24032269)
Supplement: Supplementary file 1 [file ijms-24-02269-s001.zip › Table S6.pdf]

Table S6. Three-way ANOVA results for the effects of tissue type (GL and WL), time point (9th and 13th day) and two PAR intensities as well as their interaction on the content of phenolic compounds found in the leaves of *P. zonale* plants. Experimental conditions were as described in Figure 4. The *Df*s are shown in the brackets (the first number represents *Df* of the main effects and their interactions and the second number is *Df* of error).

HL experiment

Trait	Source of variation	*F*	*P > F*	Trait	Source of variation	*F*	*P > F*
**Val**	**tissue**	**11.38**	**0.0010**	**Ala**	tissue	1.07	0.305
(*Df*: 1; 69)	**time**	**31.58**	**< 0.0001**	(*Df*: 1; 69)	**time**	**17.71**	**< 0.0001**
	**PAR**	**16.36**	**< 0.0001**		**PAR**	**57.06**	**< 0.0001**
	**tissue × time**	**16.70**	**< 0.0001**		**tissue × time**	**5.38**	**0.0070**
	tissue × PAR	3.54	0.0650		**tissue × PAR**	**6.01**	**0.0170**
	**time × PAR**	**15.77**	**< 0.0001**		**time × PAR**	**9.75**	**0.0030**
	**tissue × time × PAR**	**14.57**	**< 0.0001**		tissue × time × PAR	0.08	0.7780
**Ile**	**tissue**	**12.81**	**0.0010**	**Leu**	**tissue**	**17.70**	**< 0.0001**
(*Df*: 1; 69)	**time**	**4.96**	**0.0100**	(*Df*: 1; 69)	**time**	**4.93**	**0.0100**
	**PAR**	**4.10**	**0.0470**		**PAR**	**4.64**	**0.0350**
	**tissue × time**	**3.34**	**0.0420**		tissue × time	0.23	0.7950
	tissue × PAR	0.84	0.3640		tissue × PAR	0.71	0.4020
	time × PAR	2.61	0.1110		time × PAR	0.28	0.6010
	tissue × time × PAR	2.72	0.1040		tissue × time × PAR	2.01	0.1610
**GABA**	**tissue**	**10.01**	**0.0020**	**Pro**	**tissue**	**10.98**	**0.0020**
(*Df*: 1; 69)	time	1.82	0.1700	(*Df*: 1; 69)	**time**	**7.13**	**0.0020**
	**PAR**	**7.11**	**0.0100**		PAR	2.58	0.1130
	tissue × time	1.95	0.1510		**tissue × time**	**4.58**	**0.0140**
	tissue × PAR	0.00	0.9880		tissue × PAR	0.02	0.8780
	time × PAR	2.40	0.1260		time × PAR	2.12	0.1500
	tissue × time × PAR	0.00	0.9930		tissue × time × PAR	2.20	0.1430
**Met**	**tissue**	**129.54**	**< 0.0001**	**Ser**	**tissue**	**27.98**	**< 0.0001**
(*Df*: 1; 69)	**time**	**5.24**	**0.0080**	(*Df*: 1; 69)	**time**	**23.58**	**< 0.0001**
	PAR	3.03	0.0870		**PAR**	**11.24**	**0.0010**
	**tissue × time**	**8.92**	**< 0.0001**		**tissue × time**	**7.07**	**0.0020**
	tissue × PAR	0.19	0.6610		**tissue × PAR**	**6.86**	**0.0110**
	time × PAR	0.13	0.7160		time × PAR	1.10	0.2990
	tissue × time × PAR	0.08	0.7730		**tissue × time × PAR**	**6.97**	**0.0110**
**Thr**	**tissue**	**51.45**	**< 0.0001**	**Phe**	tissue	2.34	0.1310
(*Df*: 1; 69)	**time**	**30.28**	**< 0.0001**	(*Df*: 1; 69)	time	1.49	0.2330
	**PAR**	**15.29**	**< 0.0001**		PAR	3.30	0.0740
	**tissue × time**	**18.16**	**< 0.0001**		tissue × time	0.95	0.3920
	tissue × PAR	2.20	0.1440		tissue × PAR	1.68	0.2010
	time × PAR	1.19	0.2800		time × PAR	0.06	0.8060
	tissue × time × PAR	1.06	0.3070		tissue × time × PAR	0.00	0.9880

**Asp**	**tissue**	**219.34**	**< 0.0001**	**Glu**	tissue	0.58	0.4510
(*Df*: 1; 69)	time	2.62	0.0810	(*Df*: 1; 69)	**time**	**8.20**	**0.0010**
	**PAR**	**6.56**	**0.0130**		**PAR**	**7.67**	**0.0070**
	tissue × time	2.07	0.1350		tissue × time	3.06	0.0550
	tissue × PAR	1.32	0.2550		tissue × PAR	2.85	0.0970
	time × PAR	1.81	0.1840		time × PAR	0.49	0.4850
	tissue × time × PAR	0.02	0.8800		tissue × time × PAR	1.62	0.2080
**Asn**	**tissue**	**76.48**	**< 0.0001**	**Gln**	**tissue**	**24.95**	**< 0.0001**
(*Df*: 1; 69)	**time**	**20.93**	**< 0.0001**	(*Df*: 1; 69)	**time**	**16.37**	**< 0.0001**
	PAR	0.16	0.6960		PAR	0.86	0.3570
	**tissue × time**	**24.98**	**< 0.0001**		**tissue × time**	**11.96**	**< 0.0001**
	tissue × PAR	0.56	0.4560		**tissue × PAR**	**4.35**	**0.0410**
	time × PAR	0.82	0.3690		**time × PAR**	**4.36**	**0.0410**
	tissue × time × PAR	0.39	0.5350		**tissue × time × PAR**	**5.45**	**0.0230**
**Tyr**	**tissue**	**7.03**	**0.0100**	**Gly**	tissue	1.09	0.3030
(*Df*: 1; 69)	time	2.88	0.0640	(*Df*: 1; 60)	time	1.50	0.2340
	PAR	1.87	0.1770		PAR	0.06	0.8140
	tissue × time	0.50	0.6110		tissue × time	2.11	0.1320
	tissue × PAR	1.32	0.2550		tissue × PAR	0.09	0.7650
	time × PAR	3.67	0.0600		time × PAR	0.66	0.4210
	tissue × time × PAR	1.53	0.2210		tissue × time × PAR	0.47	0.4980
**Arg**	**tissue**	**24.37**	**< 0.0001**	**Cys**	**tissue**	**6.70**	**0.0120**
(*Df*: 1; 69)	time	1.71	0.1900	(*Df*: 1; 69)	**time**	**4.53**	**0.0150**
	PAR	0.91	0.3450		**PAR**	**4.41**	**0.0400**
	tissue × time	0.73	0.4850		tissue × time	1.96	0.1500
	tissue × PAR	0.00	0.9490		tissue × PAR	1.19	0.2790
	time × PAR	3.08	0.0850		time × PAR	1.40	0.2420
	tissue × time × PAR	2.34	0.1310		tissue × time × PAR	1.00	0.3210
**Total AAs**	**tissue**	**98.53**	**< 0.0001**				
(*Df*: 1; 69)	**time**	**17.93**	**< 0.0001**				
	**PAR**	**25.11**	**< 0.0001**				
	**tissue × time**	**10.33**	**0.0001**				
	tissue × PAR	0.03	0.8668				
	time × PAR	0.07	0.7987				
	tissue × time × PAR	0.03	0.8625				

COLD + HL experiment

Trait	Source of variation	*F*	*P* > *F*	Trait	Source of variation	*F*	*P* > *F*
**Val**	**tissue**	**49.85**	**< 0.0001**	**Ala**	**tissue**	**12.15**	**0.0010**
(*Df*: 1; 69)	**time**	**10.32**	**< 0.0001**	(*Df*: 1; 69)	time	0.25	0.7770
	**PAR**	**6.61**	**0.0130**		PAR	1.91	0.1720
	**tissue × time**	**11.67**	**< 0.0001**		**tissue × time**	**4.18**	**0.0200**
	**tissue × PAR**	**6.74**	**0.0120**		tissue × PAR	0.01	0.9120
	**time × PAR**	**17.58**	**< 0.0001**		time × PAR	3.09	0.0840

	**tissue × time × PAR**	**14.01**	**< 0.0001**		tissue × time × PAR	2.85	0.0970
**Ile**	**tissue**	**106.83**	**< 0.0001**	**Leu**	**tissue**	**140.68**	**< 0.0001**
(*Df*: 1; 69)	time	0.38	0.6850	(*Df*: 1; 69)	**time**	**9.95**	**< 0.0001**
	**PAR**	**71.53**	**< 0.0001**		**PAR**	**37.65**	**< 0.0001**
	**tissue × time**	**6.10**	**0.0040**		**tissue × time**	**14.02**	**< 0.0001**
	**tissue × PAR**	**34.61**	**< 0.0001**		**tissue × PAR**	**39.16**	**< 0.0001**
	time × PAR	**4.50**	**0.0380**		**time × PAR**	**35.07**	**< 0.0001**
	tissue × time × PAR	**21.67**	**< 0.0001**		**tissue × time × PAR**	**37.43**	**< 0.0001**
**GABA**	tissue	3.83	0.0600	**Pro**	**tissue**	**15.82**	**< 0.0001**
(*Df*: 1; 39)	**time**	**7.12**	**0.0030**	(*Df*: 1; 69)	**time**	**3.89**	**0.0260**
	**PAR**	**15.34**	**< 0.0001**		PAR	0.69	0.4110
	**tissue × time**	**17.55**	**< 0.0001**		**tissue × time**	**4.80**	**0.0120**
	**tissue × PAR**	**15.92**	**< 0.0001**		tissue × PAR	2.43	0.1250
	**time × PAR**	**10.91**	**0.0020**		time × PAR	0.57	0.4520
	**tissue × time × PAR**	**19.69**	**< 0.0001**		tissue × time × PAR	0.94	0.3360
**Met**	**tissue**	**78.51**	**< 0.0001**	**Ser**	**tissue**	**74.00**	**< 0.0001**
(*Df*: 1; 69)	**time**	**18.21**	**< 0.0001**	(*Df*: 1; 69)	time	2.68	0.0770
	**PAR**	**37.63**	**< 0.0001**		PAR	0.04	0.8350
	**tissue × time**	**21.08**	**< 0.0001**		tissue × time	2.24	0.1150
	**tissue × PAR**	**34.77**	**< 0.0001**		tissue × PAR	0.56	0.4580
	**time × PAR**	**22.08**	**< 0.0001**		time × PAR	2.31	0.1340
	**tissue × time × PAR**	**24.20**	**< 0.0001**		tissue × time × PAR	0.62	0.4350
**Thr**	**tissue**	**93.55**	**< 0.0001**	**Phe**	**tissue**	**70.27**	**< 0.0001**
(*Df*: 1; 69)	time	2.91	0.0620	(*Df*: 1; 69)	**time**	**7.29**	**0.0010**
	PAR	0.01	0.9160		**PAR**	**13.26**	**0.0010**
	tissue × time	3.15	0.0500		**tissue × time**	**6.29**	**0.0030**
	tissue × PAR	0.76	0.3870		**tissue × PAR**	**26.15**	**< 0.0001**
	time × PAR	2.23	0.1410		time × PAR	0.14	0.7110
	**tissue × time × PAR**	**8.11**	**0.0060**		tissue × time × PAR	0.00	0.9610
**Asp**	**tissue**	**122.14**	**< 0.0001**	**Glu**	tissue	3.13	0.0820
(*Df*: 1; 69)	time	2.30	0.1090	(*Df*: 1; 69)	**time**	**4.28**	**0.0180**
	PAR	0.43	0.5140		PAR	1.60	0.2110
	tissue × time	1.10	0.3380		tissue × time	0.40	0.6730
	tissue × PAR	0.22	0.6390		tissue × PAR	1.45	0.2330
	**time × PAR**	**8.90**	**0.0040**		**time × PAR**	**5.42**	**0.0230**
	**tissue × time × PAR**	**8.58**	**0.0050**		**tissue × time × PAR**	**11.04**	**0.0020**
**Asn**	**tissue**	**98.29**	**< 0.0001**	**Gln**	**tissue**	**14.96**	**< 0.0001**
(*Df*: 1; 69)	**time**	**10.018**	**< 0.0001**	(*Df*: 1; 69)	time	1.59	0.2130
	PAR	3.65	0.0610		**PAR**	**6.12**	**0.0160**
	**tissue × time**	**10.386**	**< 0.0001**		**tissue × time**	**11.21**	**< 0.0001**
	tissue × PAR	3.141	0.0810		tissue × PAR	0.79	0.3770
	**time × PAR**	**4.016**	**0.0500**		time × PAR	**16.36**	**< 0.0001**

	tissue × time × PAR	3.515	0.0660		tissue × time × PAR	2.28	0.1360
**Tyr**	**tissue**	**54.45**	**< 0.0001**	**Gly**	tissue	**54.53**	**< 0.0001**
(*Df*: 1; 59)	**time**	**46.64**	**< 0.0001**	(*Df*: 1; 69)	time	**6.05**	**0.0040**
	PAR	2.87	0.0960		PAR	2.83	0.0980
	**tissue × time**	**28.77**	**< 0.0001**		tissue × time	**5.40**	**0.0070**
	tissue × PAR	0.48	0.4900		tissue × PAR	**8.36**	**0.0050**
	time × PAR	3.47	0.0690		time × PAR	1.08	0.3030
	tissue × time × PAR	1.42	0.2400		tissue × time × PAR	0.05	0.8250
**Arg**	**tissue**	**124.59**	**< 0.0001**	**Lys**	tissue	**334.46**	**< 0.0001**
(*Df*: 1; 69)	**time**	**9.33**	**< 0.0001**	(*Df*: 1; 49)	time	**16.97**	**< 0.0001**
	PAR	3.28	0.0750		PAR	**19.63**	**< 0.0001**
	**tissue × time**	**9.55**	**< 0.0001**		tissue × time	**25.78**	**< 0.0001**
	tissue × PAR	3.43	0.0690		tissue × PAR	**37.77**	**< 0.0001**
	time × PAR	1.10	0.2980		time × PAR	**22.73**	**< 0.0001**
	tissue × time × PAR	1.46	0.2320		tissue × time × PAR	3.93	0.0540
**Trp**	tissue	**6.44**	**0.0140**	**Total AAs**	**tissue**	**253.00**	**< 0.0001**
(*Df*: 1; 64)	time	1.41	0.2540	(*Df*: 1; 69)	**time**	**6.67**	**0.0024**
	PAR	3.48	0.0680		PAR	0.37	0.5452
	tissue × time	1.42	0.2510		**tissue × time**	**5.97**	**0.0043**
	tissue × PAR	3.70	0.0600		tissue × PAR	3.50	0.0663
	time × PAR	2.77	0.1020		**time × PAR**	**9.78**	**0.0027**
	tissue × time × PAR	2.93	0.0930		**tissue × time × PAR**	**8.02**	**0.0063**
