# Supplementary material for: Carbon and Nitrogen Allocation between the Sink and Source Leaf Tissue in Response to the Excess Excitation Energy Conditions
Source: Int J Mol Sci. 2023 Jan 23;24(3):2269. doi: 10.3390/ijms24032269 (PMC9917124; doi:10.3390/ijms24032269)
Supplement: Supplementary file 1 [file ijms-24-02269-s001.zip › Table S7.pdf]

Table S7. Sequences of forward and reverse primers used for the detection of selected transcripts.

Gene	Forward primer	Reverse primer
POD17	CCGAATCCATTGTCCGGGAT	CCTCCTTAGCTTCGTCGACC
POD42	ACCCGGGTCTTGTCATGAAC	CTCTCTCTCCACAGCCTCCT
POD3	TGGGCAAGATGGGTTGTGTT	ACAGCACTGAGGCATCACAA
MDAR	GAGGTGGAAATTCGGCTGGA	ACAAGTGTGGAAACCAGGCA
DFR	CAAGCGGCTTGGAAGTATGC	GGCTTTGGGGTGCTCATACA
Actin	AGCTGAGAGATTCCGTTGCC	CCTTGCTCATCCTGTCAGCA
CHS	GCCTTCGAGCCTTTGGGTAT	CAGCCCATCCTCACACGATT
PAL	ACTTGGAGGAAGTGAAGCGG	ATCACTGCTCGCCTTCACTC
ANS	AACCCTAACAAGTGGCCCAC	GGCTGAGGGCATTTTGGGTA
AGXT	AGTGTGAAGAAAGCGGGAGG	TGCCAGTGTCACTATGTCCG

POD, class III peroxidase; MDAR, monodehydroascorbate reductase; DFR, dihydroflavonol 4-reductase; CHS, chalcone synthase; PAL, phenylalanine ammonia lyase; ANS, anthocyanidin synthase; AGXT, alanine-glyoxylate aminotransferase;
