# Supplementary figures and images for: Carbon and Nitrogen Allocation between the Sink and Source Leaf Tissue in Response to the Excess Excitation Energy Conditions
Source: Int J Mol Sci. 2023 Jan 23;24(3):2269. doi: 10.3390/ijms24032269 (PMC9917124; doi:10.3390/ijms24032269)

5 day

7 day

9day

11 day

13 day

**A) Wrapped leaves**

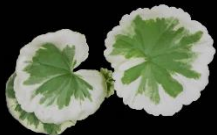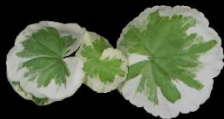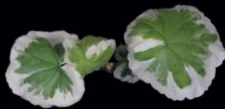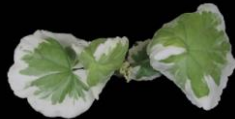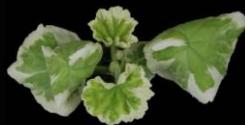

**B) Accumulation of anthocyanins**

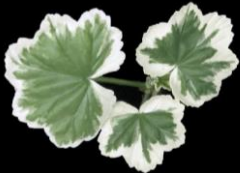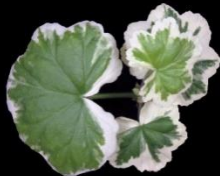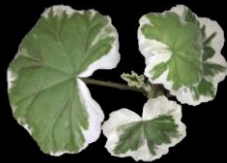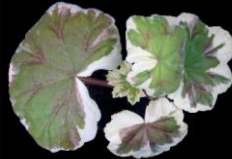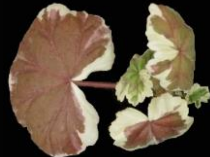

Supplement: Supplementary file 1 [file ijms-24-02269-s001.zip › Figure S1.pdf]

HL

COLD+HL

G

W

G

W

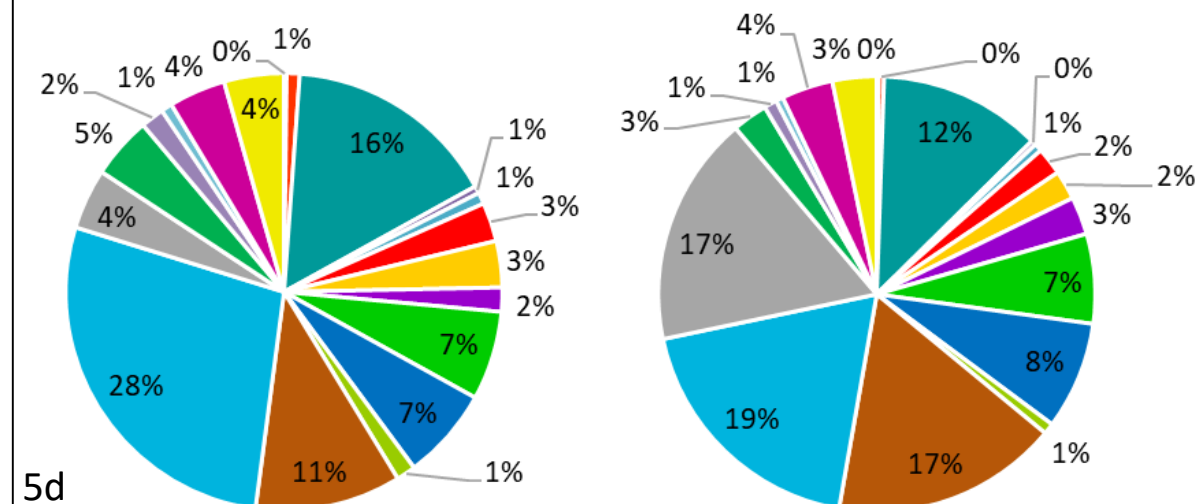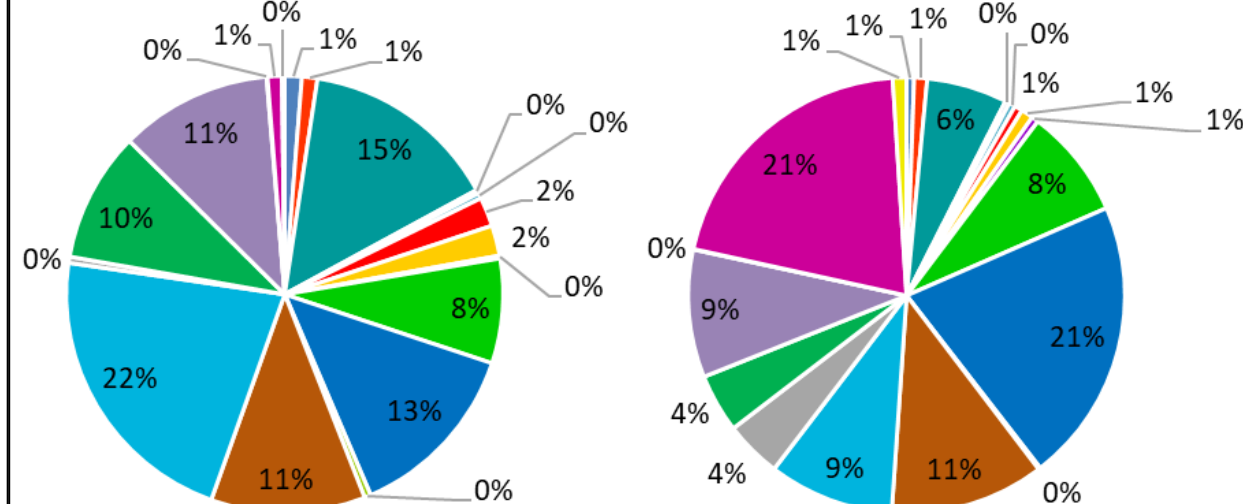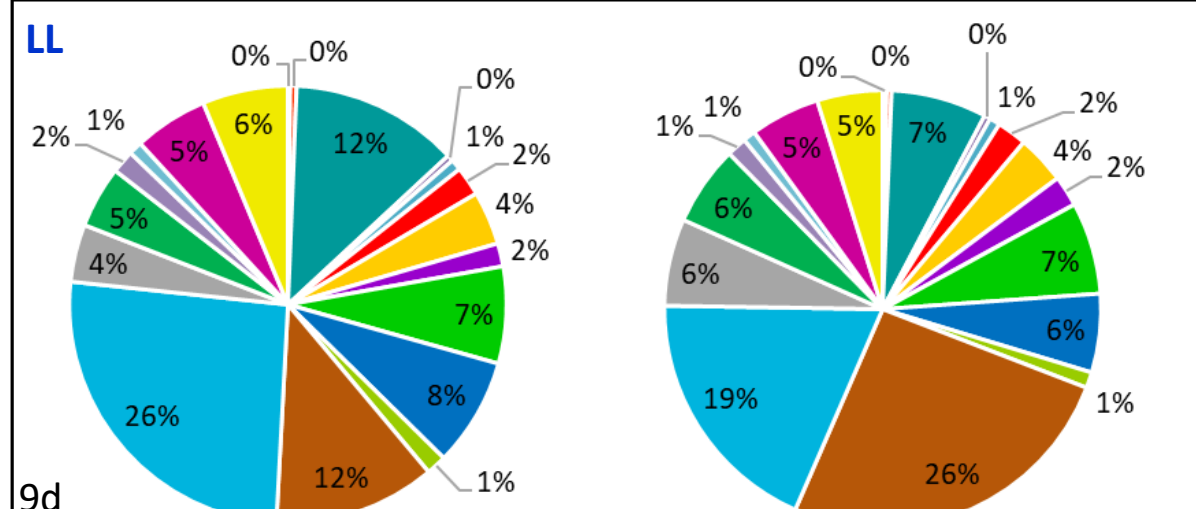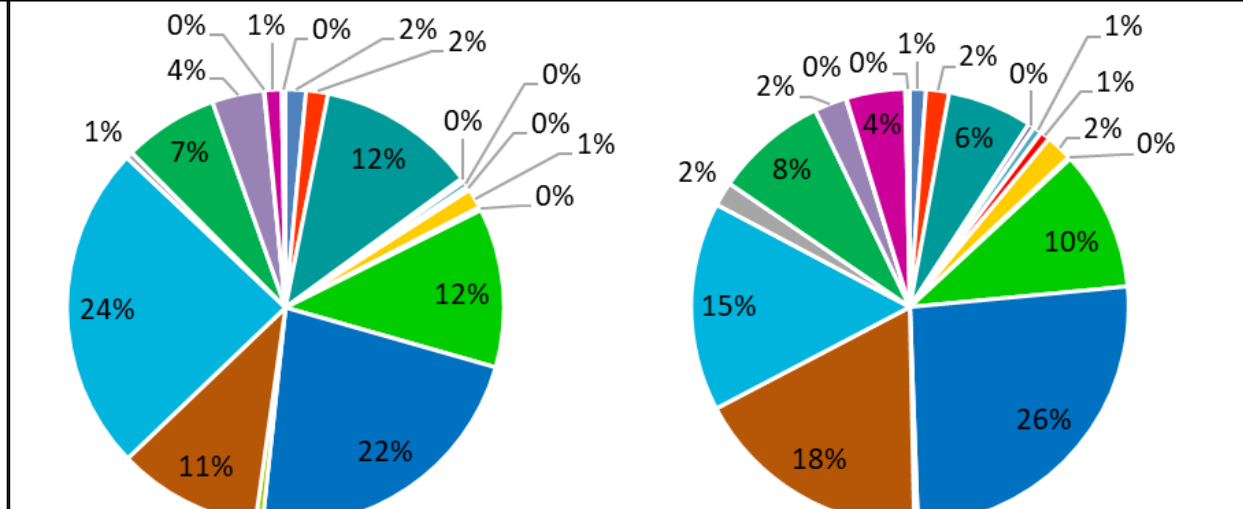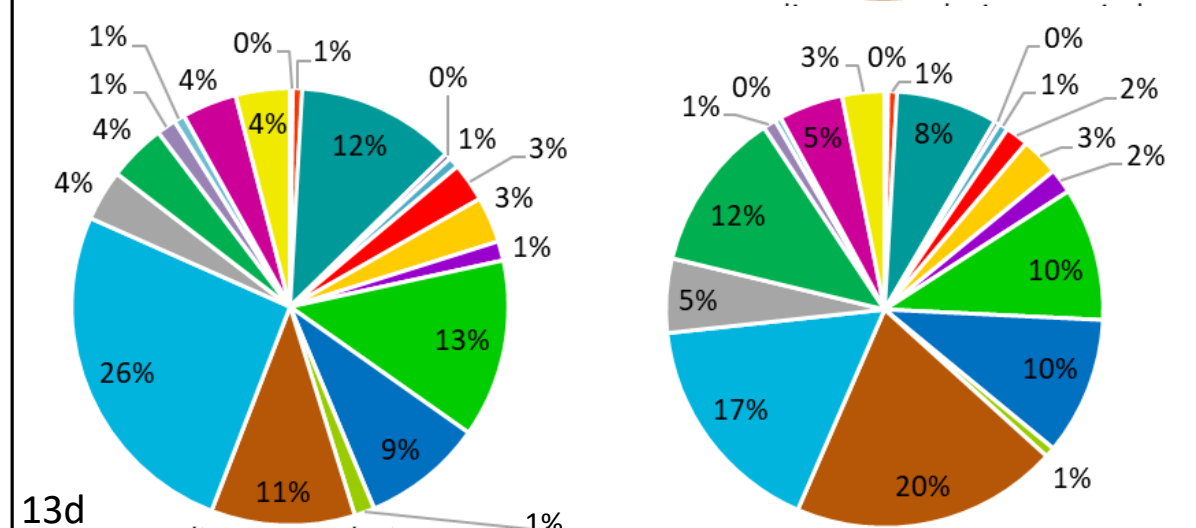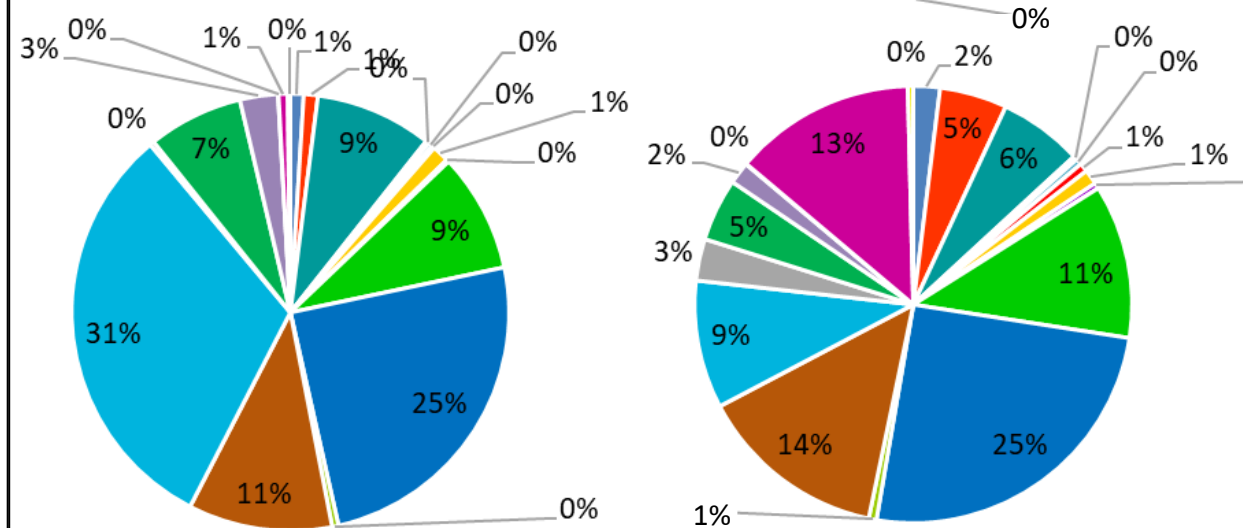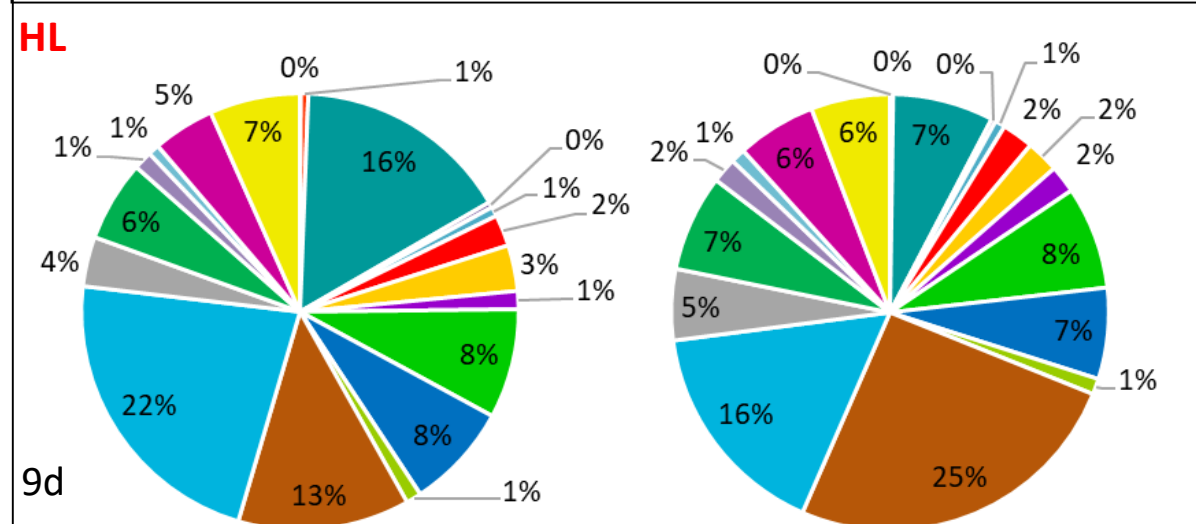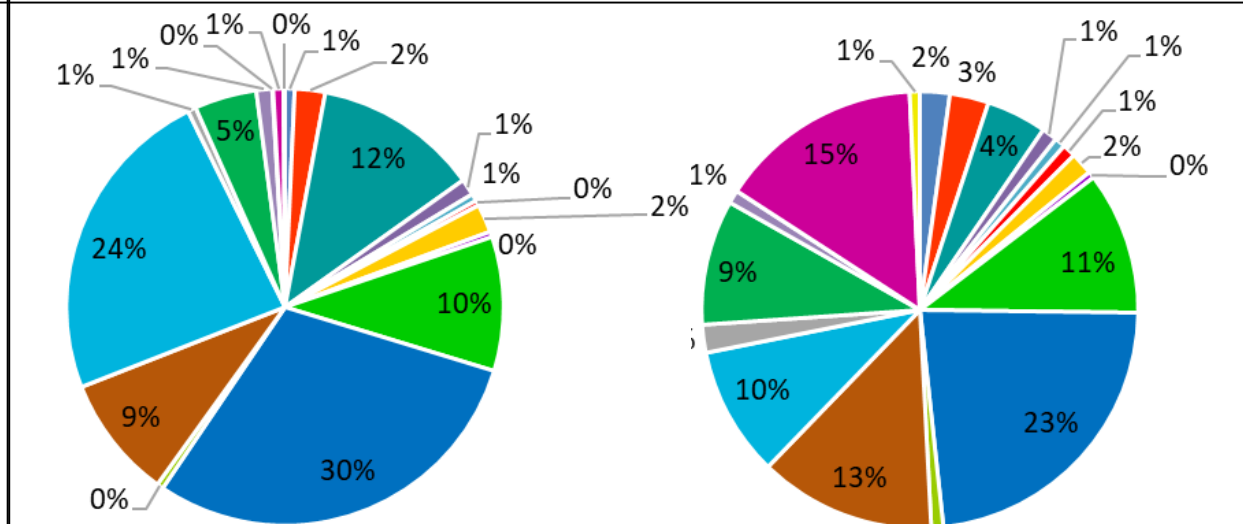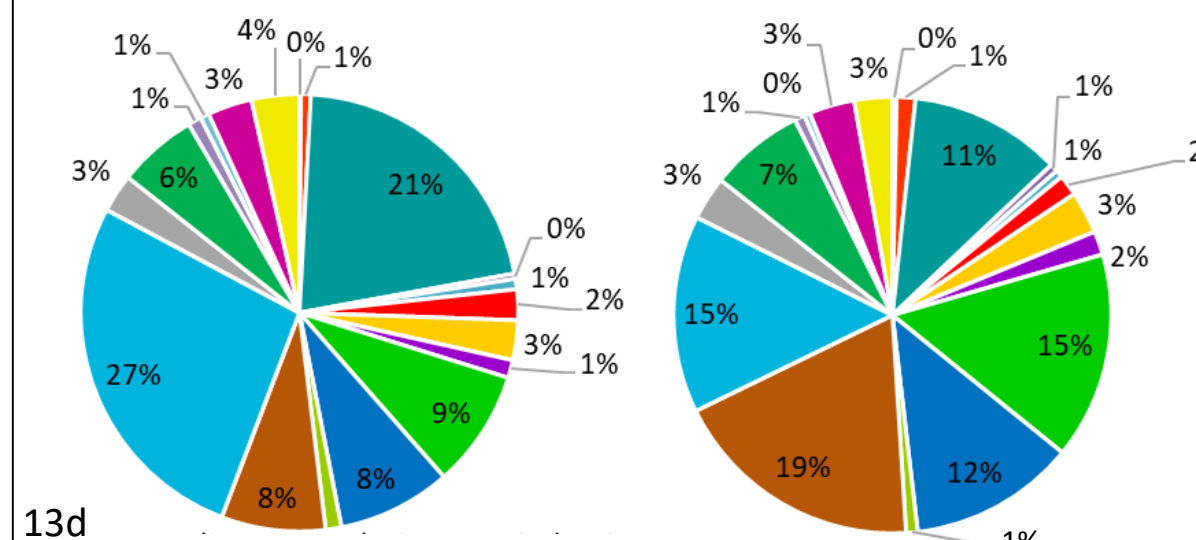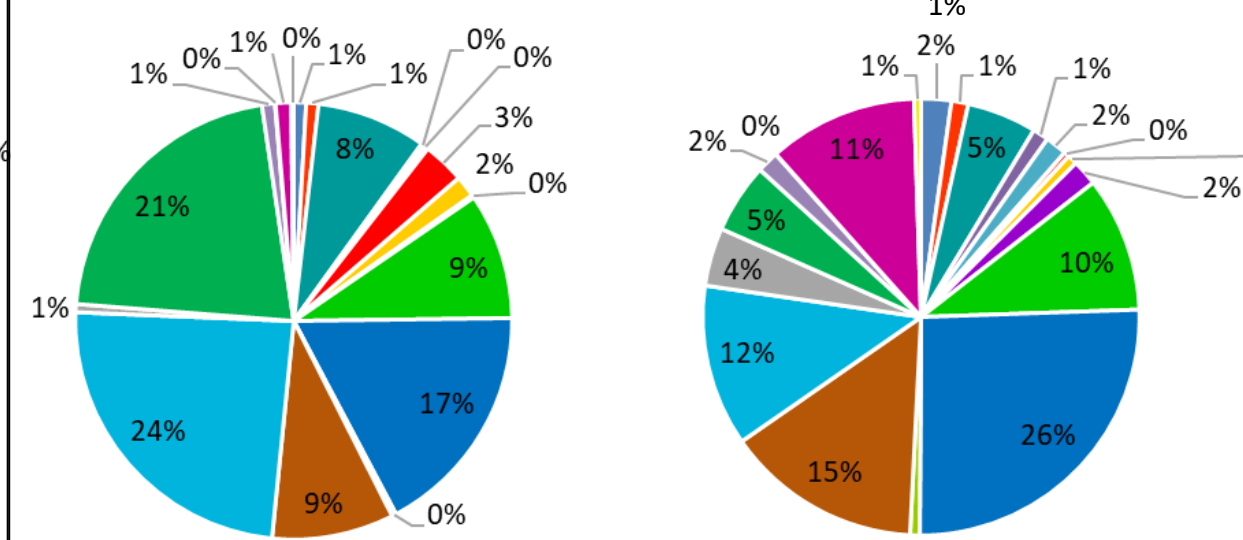

Gly Val Ala Ile Leu GABA Pro Met Ser Thr  
 Phe Asp Glu Asn Gln Tyr Cys Arg Trp

Supplement: Supplementary file 1 [file ijms-24-02269-s001.zip › Figure S2.pdf]
